# Molecularly Imprinted Polymers as Extracting Media for the Chromatographic Determination of Antibiotics in Milk

**DOI:** 10.3390/molecules23020316

**Published:** 2018-02-02

**Authors:** Dimitrios Bitas, Victoria Samanidou

**Affiliations:** Laboratory of Analytical Chemistry, Department of Chemistry, Aristotle University of Thessaloniki, 54124 Thessaloniki, Greece; dimitriosbitas@gmail.com

**Keywords:** molecularly imprinted polymers, MIPs, antibiotics, milk, extraction, chromatography

## Abstract

Milk-producing animals are typically kept stationary in overcrowded large-scale farms and in most cases under unsanitary conditions, which promotes the development of infections. In order to maintain sufficient health status among the herd or promote growth and increase production, farmers administer preventative antibiotic doses to the animals through their feed. However, many antibiotics used in cattle farms are intended for the treatment of bacterial infections in humans. This results in the development of antibiotic-resistant bacteria which pose a great risk for public health. Additionally, antibiotic residues are found in milk and dairy products, with potential toxic effects for the consumers. Hence the need of antibiotic residues monitoring in milk arises. Analytical methods were developed for the determination of antibiotics in milk, with key priority given to the analyte extraction and preconcentration step. Extraction can benefit from the production of molecularly imprinted polymers (MIPs) that can be applied as sorbents for the extraction of specific antibiotics. This review focuses on the principals of molecular imprinting technology and synthesis methods of MIPs, as well as the application of MIPs and MIPs composites for the chromatographic determination of various antibiotic categories in milk found in the recent literature.

## 1. Introduction

Milk-producing animals are typically kept overcrowded under unsanitary conditions suffering from various infections and for this reason they are administered antibiotics through their feed or water. Dairy cows are treated with antibiotics in therapeutic doses in order to treat diseases such as mastitis, bacteremia, diarrhea and pulmonary diseases or for prophylactic reasons. They are also administered at sub-therapeutic doses for growth promoting purposes. However, the excessive use of antibiotics in human and veterinary medicine has resulted in the development of antibiotic-resistant bacteria. Furthermore, antibiotic residues are found in milk and dairy products, posing a threat for human health, since they can cause allergic reactions and other toxic effects to sensitive groups. For this reason, the European Union has published the “Commission Regulation (EU) No. 37/2010 of 22 December 2009 on pharmacologically active substances and their classification regarding maximum residue limits in foodstuffs of animal origin” and every animal originated product should comply with [1,2,3].

Analytical methods were developed for the determination of antibiotic residues in milk. Key priority in any analytical method is given to the analyte clean-up and preconcentration step, especially in complex matrices such as milk. Analytical performance is dependent to a well-developed sample preparation protocol. Sample preparation approaches have already been reviewed in the literature for the extraction and determination of antibiotics in milk samples [2,4,5,6], along with other food samples [7,8].

Molecularly Imprinted Polymers (MIPs) are synthetic polymeric materials with imprinted sites complementary to a specific molecule and high affinity over analytes with analogous molecular structure. Sample preparation protocols can benefit from the selectivity of the MIPs over specific molecule or group of molecules for improved clean-up efficiency. MIPs have already been reviewed as sorbents in sample preparation techniques [9,10,11] such as solid-phase extraction (SPE) [12,13,14], solid-phase microextraction (SPME) [15,16] and on-line extraction techniques [17] for bioanalysis [18], pharmaceutical [19], environmental [20], food [21], and forensic analysis [22]. MIPs have also been reviewed for the extraction of non-steroidal anti-inflammatory drugs and analgesics [23], drugs of abuse [24], personal care products [25] and proteins [26]. Apart from sample preparation purposes, MIPs have already been reviewed as monolithic columns in high-performance liquid chromatography (HPLC) and capillary electrophoresis (CE) [27,28], in biosensors [29] and gas sensors [30], in drug delivery [31], for diagnostic purposes [32,33], in wastewater treatment [34] and for biomolecule identification and separation [35].

This review focuses on the basics of molecular imprinting technology and polymerization techniques, as well as the application of MIPs for the chromatographic determination of various antibiotic categories in milk found in the recent literature.

## 2. Molecular Imprinting

**Figure 1:**
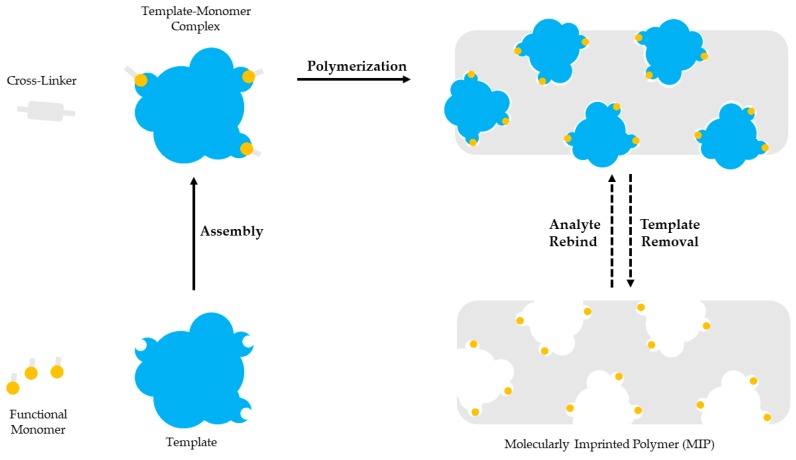
The template molecule and the functional monomer interact. The template-monomer complexes and a cross-linker polymerize. The template molecule is removed to provide a polymer with imprinted sites.

Molecular imprinting is based on the polymerization of a functional monomer and a cross-linker around a template molecule. Briefly, a selected template molecule and a functional monomer interact covalently or non-covalently and develop complexes. Then, polymerization takes place between the developed template-functional monomer complexes and a cross-linker. After the polymerization the template molecule is removed, leaving a polymer with imprinted sites complementary to the molecular structure and the functional groups of the template. The imprinted sites are available for binding the molecules with the same or similar structure as the template [36,37,38]. Molecular imprinting is schematically presented in Figure 1.

The essential components of a molecular imprinting process are the template molecule, the functional monomer and the cross-linker. The polymerization initiator and the porogenic solvent are also important. Furthermore, polymerization is conducted under a stream of an inert gas such as nitrogen to ensure oxygen removal. 

For the selection of the template molecule specific criteria should be met. The functional groups of the template should be able to interact with the functional monomers and not hinder the polymerization reaction, while the template molecule should be chemically stable during polymerization. Usually, the templates are small organic molecules, such as pesticides, pharmaceuticals, sugars, amino acids and peptides [39,40].

Functional monomers consist of a recognition unit that interacts with the template and a polymerizable unit that is polymerized with the cross-linker. The recognition site of the selected functional monomer should develop strong interactions with the functional groups of the template molecule to form a complex before polymerization takes place. Functional monomers such as acrylic acid, methacrylic acid (MAA), methyl methacrylate, 2-(trifluoromethyl)acrylic acid (TFMAA), styrene, 4-vinylpyridine (4-VP), acrylamide (AM), methacrylamide, 2-hydroxyethyl methacrylate (HEMA) and 3-aminopropyltriethoxysilane (APTES) are used in non-covalent imprinting [36,39,40].

The cross-linker polymerizes with the polymerizable unit of the functional monomer to form a highly cross-linked polymer around the template-monomer complex that remains firm after the template removal. The selectivity and the binding capacity of the developed MIP depend on the amount of the selected cross-linker. Insufficient amount of the cross-linker results in mechanically unstable poorly cross-linked MIPs, one the other hand increased amount results in MIPs with reduced imprinted sites. Cross-linkers such as ethylene glycol dimethacrylate (EGDMA), trimethylopropane methacrylate (TRIM), divinylbenzene (DVB) and tetramethylene dimethacrylate are used in non-covalent imprinting [36,39,40].

The selection of the porogenic solvent is also important in molecular imprinting. The porogen influences the interaction between the template and the monomer, serves as the dispersion media and affects the pore forming during the polymerization reaction. In non-covalent imprinting less polar or non-polar solvents such as toluene, acetonitrile and chloroform are preferred [39,40].

Most MIPs are prepared by free radical polymerization that is initiated either thermally or photochemically with the addition of a peroxy or an azo compound. The most frequently used azo compound is azobisisobutyronitrile (AIBN) that is used at temperature range of 50–70 °C [39].

After the polymerization reaction is complete, template removal is essential before the prepared MIPs can be used in any application. Template removal should be meticulously performed in order to ensure that the maximum number of imprinted sites are free of the template molecule, especially in analytical applications where template bleeding can affect the results. Complete removal of the template often requires extreme conditions that can impair the imprinted sites. Template removal can be performed by three main approaches, solvent extraction, physically assisted extraction or the use of subcritical/supercritical solvents (subcritical water/supercritical CO_2_). Physically assisted extraction includes ultrasound-assisted, microwave-assisted and pressurized liquid extraction. The most common approach is the use of organic solvents, either by incubating the prepared MIPs in organic solvents or by extraction in a Soxhlet apparatus. MIP incubation is a mild template removal method that requires several hours to be complete but does not impair the imprinted sites. Incubation in organic solvents causes swelling of the MIP structure and favors template removal, while heating and stirring can reduce the duration of template removal. Extraction in a Soxhlet apparatus is a more drastic method where the template is extracted for several hours with heated organic solvent and is more commonly used for MIPs prepared by bulk polymerization. The heated solvent increases template solubility, thus template removal. However, Soxhlet extraction is a time-consuming method (up to 24 h) that requires increased volumes of organic solvent and high temperatures can cause template degradation [41].

There are two main approaches for the preparation of MIPs based on the interactions between the template molecule and the functional monomer that can be either covalent or non-covalent. In the covalent approach, reversible covalent interactions are developed between the functional groups of the template and the recognition unit of the monomer. Covalent imprinting is stoichiometric and functional monomer residues can only be found in the imprinted sites. Template removal is usually achieved by treating the prepared MIPs with organic solvents or Soxhlet extraction, while molecule binding is based on covalent interactions. However, there are limited reversible covalent interactions available and the strong nature of covalent interactions does not favor fast binding and removal on the imprinted sites [38,40,42].

In the non-covalent approach, hydrogen bonding, van der Waals forces and ionic or π-π interactions are developed for the formation of the template-monomer complex in a pre-polymerization step, with hydrogen bonding being the most common interaction. Template removal is achieved by washing the prepared MIPs with an organic solvent or a mixture of solvents, while molecule binding is based on non-covalent interactions. Non-covalent imprinting is a simple MIP preparation process that offers fast binding and removal on the imprinted sites and is the most preferred approach. However, the interactions forming the template-monomer are not as stable and prone to disruption [38,40,42].

Classic molecular imprinting involves a single template for the preparation of a MIP with imprinted sites that are not able to recognize more than one target molecule. With multi-template imprinting more templates are used for the preparation of MIPs with more than one type of imprinted sites able for simultaneous recognition of multiple target molecules. The multi-template MIPs enable the extraction and determination of multiple analytes; however, they have reduced selectivity compared with one-template MIPs [39].

Like multi-template imprinting, MIPs can be prepared by employing more than one functional monomer. Each functional monomer is able to recognize a different functional group of the template molecule and the resulting in MIPs presents increased selectivity. Multi-functional monomer imprinting can be helpful for the imprinting of macromolecules. However, thorough study is required for the selection of the appropriate monomers and their combining synergy in preparing MIPs with multiple functional monomers [39]. 

Sometimes in molecular imprinting the desirable template molecule can be chemically unstable or has low solubility during polymerization, difficult to handle, or even an expensive compound, thus dummy imprinting employs a surrogate structurally analogue molecule as an alternative template for MIP preparation. Furthermore, the drawback of template bleeding when MIPs are used for SPE applications can be removed by the employment of dummy imprinted MIPs [36,38,39].

### MIP Characterization

Morphological evaluation of the prepared MIPs can be accomplished by scanning electron microscopy (SEM) and transmission electron microscopy (TEM), while structure analysis by X-ray photoelectron spectroscopy (XPS), extended X-ray absorption fine structures (EXAFS) and X-ray diffraction studies. Template molecule-functional monomer complex interactions can be screened by infrared (IR), nuclear magnetic resonance (NMR) and UV-Vis spectroscopy. In the case of MMIPs, the magnetic properties of MMIPs can be analyzed by vibrating sample magnetometer (VSM) [36,39].

In addition to the MIP, a non-imprinted polymer (NIP) is usually prepared without the presence of the template molecule. Although NIP has no imprinted sites, NIP preparation is carried out under the same conditions as the MIP and interactions between the NIP and the target molecule can be observed. This acts as control to characterize the quality of imprinted sites on the MIP surface and measure how strong are the interactions between the MIP and the target molecule in comparison with those between the NIP and the template. Binding tests are conducted by applying both the prepared MIP and NIP in solutions of predetermined concentration of the target molecule and measuring the retained amount. Binding tests can be conducted in solutions consisting of the same solvent used in MIP preparation or a solvent that simulates the nature of the analyzed sample. By doing these tests, the best type of functional monomer or template/monomer ratio can be chosen to optimize MIP preparation, while the best solvent for analyte extraction or analyte elution can be selected to optimize the sample preparation protocol [36].

## 3. Polymerization Techniques for MIP Preparation

There are two main approaches applied for the preparation of MIPs, free radical polymerization, controlled radical polymerization and sol-gel process.

### 3.1. Free Radical Polymerization (FRP)

**Figure 2:**
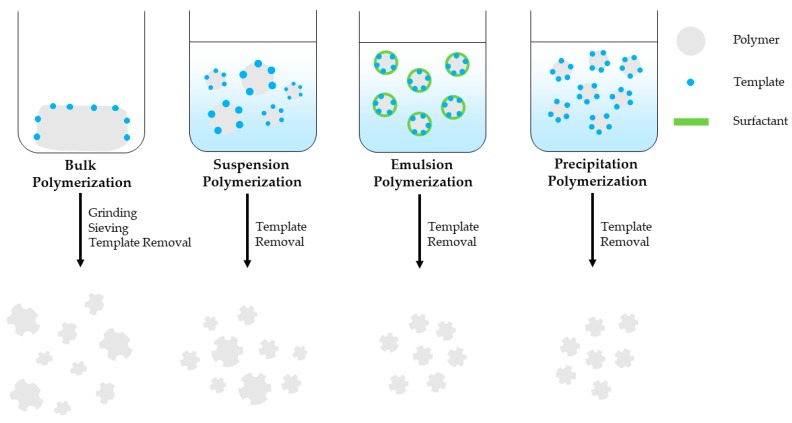
Free radical polymerization techniques used for MIP preparation.

Bulk polymerization is a fast and simple polymerization method that provides pure MIPs with no special instrumentation requirements. It is the most widely used free radical polymerization method for preparing MIPs applied in sample extraction techniques. However, this method requires increased template amount and the prepared MIP bulk should be grinded, sieved and sedimented with the use of a solvent in order to remove the finer particles and obtain particles of the preferred size. This is time consuming and the resulting particles have irregular shape and size, decreased number of imprinted sites and binding capacity, while template bleeding can be observed [36,37,38,39]. Suspension polymerization is a simple, single-step polymerization method where polymerization mixture is suspended in a continuous aqueous, mineral oil or perfluorocarbon liquid phase and provides spherical porous MIP particles. However, the developed MIP particles size ranges between μm-mm with decreased recognition capability, unsuitable for solid-phase extraction applications [38,39]. Emulsion polymerization takes place in an oil/water diphasic system with the addition of surfactants that prevents diffusion and favors the formation of small, homogeneous emulsion droplets. Emulsion polymerization can provide mono-dispersed MIP particles, in high yield, however surfactant residues can interfere with the imprinted sites, thus reduce binding capacity of the MIPs [37,38,39]. Precipitation polymerization is a single-step polymerization method that provides high-quality, spherical MIP particles with homogenous size in good yield. As the polymerization takes place spherical MIP particles precipitate from the reaction solution. However, this method requires increased amount of organic solvent and meticulous control over the polymerization conditions such as solvent polarity, temperature and stirring speed that affect the MIP particle size [36,37,38,40]. MIP monoliths can be prepared inside a confined space such as a chromatographic column by in-situ polymerization. It is a simple, one-stem process and unlike bulk polymerization it does not require grinding and sieving of the prepared MIP bulk [38,39,40]. MIPS prepared by bulk, suspension, emulsion and precipitation polymerization are schematically presented in Figure 2.

### 3.2. Controlled Radical Polymerization (CRP)

Polymerization usually involves reaction initiation, polymer chain propagation and reaction termination. In free radical polymerization, reaction initiation is slow while chain propagation is fast. Additionally, side reactions such as chain transfer reactions between the components of the polymerization mixture or reactions with impurities can occur. As a result, polymerization is prematurely terminated and polymer chains with variable lengths are formed. For this reason, MIP size, structure and molecular weight cannot be controlled, while heterogenous cross-linked network and imprinted site distribution can be observed. Controlled radical polymerization offers control over the polymerization reaction with the use of capping agents that prevent premature termination. Reaction initiation is fast, while chain propagation is slow and simultaneous for all polymer chains. The resulting polymer chains have homogenously distributed size and molecular weight. 

The most common controlled radical polymerization approaches are atom transfer radical polymerization (ATRP), nitroxide-mediated polymerization (NMP) and reversible addition-fragmentation chain transfer (RAFT) polymerization. ATRP involves a reversible redox reaction catalyzed by a metal-ligand complex and the resulting MIPs have functional groups that enable surface modification. However, ATRP uses in MIP synthesis are limited because the functional groups of the template molecule and the recognition unit of the functional monomer can inhibit the metal-ligand complex. NMP involves a thermally reversible termination reaction that produces a nitroxyl radical that allows control over the polymerization reaction. Although NMP can be used in the presence of various functional groups, it requires temperatures over 100 °C that can affect the non-covalent interactions between the template molecule and the functional monomer, thus it is limited to covalent imprinting approaches. RAFT polymerization involves reversible addition-fragmentation sequences initiated by a FRP initiator and chain propagation is achieved with the help of a chain transfer agent. It can be used with a variety of functional groups and is suitable for non-covalent imprinting purposes [43,44].

#### Surface Imprinting

In surface imprinting, the template molecule is immobilized on the surface of a suitable material, where polymerization takes place, in order to prepare MIPs with controllable imprinted sites. The template can be completely removed and the resulting imprinted sites have improved accessibility that is important for imprinting macromolecules such as proteins. However, the number of the imprinted sites is proportionate to the surface area of the substrate material [37,38,39]. 

Core-shell MIPs can be prepared by surface imprinting on nanomaterials such as chitosan, polystyrene, SiO_2_, TiO_2_, and Fe_3_O_4_ combining the properties of both MIPs and nanomaterials. For the preparation of magnetic MIPs (MMIPs), appropriately prepared Fe_3_O_4_ nanoparticles undergo functionalization or surface modification and then molecular imprinting takes place on the surface of the magnetite nanoparticles, either with a free radical polymerization method or the sol-gel process. MMIPs can be collected from a solution with the application of an external magnet, thus they can be used in sample preparation providing an easier, less time-consuming and efficient analyte extraction procedure [37,38,45,46].

### 3.3. Sol-Gel Synthesis

Highly cross-linked materials can be prepared by the sol-gel synthesis that involves the hydrolysis of a tetraalkylosilane such as tetraethoxysilane (TEOS) and tetramethoxysilane (TMOS) into a colloidal solution and the polycondensation of the solution into a silica-based material. Compared with free radical polymerization, sol-gel process can take place in room temperature, is resistant to chemical and thermal decomposition and requires non-toxic solvents such as methanol and ultrapure water Fabric [36,47]. Although the sol-gel process is convenient and results in porous MIPs, the binding-rebinding process on the imprinted sites is slow and hard to be achieved [17]. Furthermore, there are limited polymerization reactions and precursors utilized in sol-gel synthesis of MIPs [39].

## 4. MIPs in Sample Preparation

The ability of the imprinted sites to recognize and bind with a specific molecule, makes MIPs excellent sorbent materials for sample preparation techniques such as solid-phase extraction (SPE), dispersive solid-phase extraction (DSPE), matrix solid-phase dispersion (MSPD) and solid-phase microextraction (SPME).

### 4.1. Molecularly Imprinted Solid-Phase Extraction (MISPE)

Following the classic SPE approach, MIP particles are packed inside an empty cartridge. The MIP SPE cartridges are preconditioned and loaded with a sample. Before analyte elution the MIPs are washed with a solvent or a mixture of solvents such as methanol, acetonitrile or water that will remove all interferences and not the bound analyte. Elution is carried out by a solvent that is able to release the bound analyte from the imprinted sites. MIPs prepared by bulk polymerization are very common in SPE applications. Furthermore, MIP packed pre-columns or monolithic columns can be used in on-line with an analytical instrument [37,38,40].

### 4.2. Dispersive Solid-Phase Extraction (DSPE)

In DSPE, MIP particles can be dispersed directly into the sample solution and after the extraction is complete they can be collected by centrifuging or filtration. This technique requires reduced MIPs amount in comparison with SPE and offers improved contact of the sorbent with the sample components, while eliminating the tedious packing the MIPs inside an SPE cartridge and the preconditioning step [38,39].

### 4.3. Matrix Solid-Phase Dispersion (MSPD)

Most sample preparation techniques are usually applied in sample solutions that result from a sample pre-treatment step, thus they cannot be applied directly to solid, semi-solid or samples with increased viscosity. With MSPD the MIP particles are added directly to the sample, mechanically mixed and the resulting mixture is packed inside a cartridge, washed and the analytes are eluted with the appropriate solvent. This technique has decreased organic solvent requirements and reduces matrix interferences [39].

### 4.4. Solid-Phase Microextraction (SPME)

In SPME, a syringe-like instrument with a sorbent-coated fiber at the end of the needle is applied for the analyte extraction from sample solutions. MIP-coated SMPE fibers can be prepared by synthesizing MIPs on the surface of a stainless steel or silica fiber. The coated fibber can be applied directly to the sample solution and after extraction it is injected directly to the analytical instrument and the analyte is desorpted either by an appropriate organic solvent in the case of an HPLC application or thermally in the case of a GC application. Apart from MIP-coated fibers, MIP monolith fibers can be prepared and used in an SPME protocol. Polymerization is performed inside a sealed capillary and after completion a part of the capillary is removed so that the MIP monolith is exposed [37,38,40,42,46].

## 5. MIP Applications for the Extraction of Antibiotics in Milk

The following section provides information about the reported applications of MIPs for the extraction and determination of amphenicol, cephalosporin, macrolide, penicillin, quinolone, sulfonamide and tetracycline antibiotic categories in milk. Emphasis is given in both synthesis and extraction protocols followed in each report.

### 5.1. Amphenicols

Amphenicols are broad-spectrum antibiotics class that includes chloramphenicol (CAP), thiamphenicol (TAP) and florfenicol (FFC). CAP is the oldest amphenicol antibiotic and was originally isolated in 1947 from *Streptomyces venezuelae* cultures. It inhibits bacterial proteins synthesis; thus, it is effective against many bacteria strains and used for clinical purposes. CAP, TAP and FFC are used for veterinary purposes, for bacterial infection prevention and treatment. However, high toxicity and unwanted effects can be observed and their use in food-producing animals is illegal in most countries. The maximum residue limit in milk according to the Commission Regulation (EU) No. 37/2010 is 50 μg/kg for TAP, while FFC is not recommended for milk-producing animals and CAP is prohibited [1,2,3].

MIPs were reported as sorbent materials for the extraction of amphenicol antibiotics from milk samples. Synthesized MIPs were mainly employed in MISPE protocols, with reports of polymer monolith microextraction (PMME) protocols. Furthermore, commercially available MISPE cartridges were employed for the extraction of CAP.

A MISPE-liquid chromatography-mass spectrometry (LC-MS/MS) method was developed for the determination of CAP. TAP was used as a dummy template for the preparation of the MIPs. A mixture of TAP (0.25 mmol), MAA (1 mmol) and methanol (40 mL) was left for 30 min and EGDMA (5 mmol) and AIBN (30 mg) were added. The mixture was sealed and purged with nitrogen for 10 min and placed in a water bath at 60 °C for 24 h. The developed MIPs were collected and the template was removed by Soxhlet extraction with methanol-acetic acid (90:10, *v*/*v*). The MIP particles were washed with methanol and dried under vacuum at 50 °C. TAP-MIPs (30 mg) were dispersed in acetone (3 mL) and packed into a SPE cartridge, washed with methanol-acetic acid (90:10, *v*/*v*) and preconditioned with methanol (3 mL) and water (1 mL). The cartridge was loaded with milk extract (10 mL) and washed with water (1 mL). The analytes were eluted with methanol-acetic acid (90:10, *v*/*v*; 3 mL), the eluate was concentrated under nitrogen stream and re-dissolved in water. In comparison with the respective NIP, MIP adsorption capacity for CAP was higher, with maximum adsorption capacities (Q_max_) of 1188.49 and 2775.94 μg/g, while imprinting factors were 1.45 for CAP and 1.35 for TAP [48].

A MISPE-high-performance liquid chromatography-ultraviolet detection (HPLC-UV) method was developed for the determination of CAP in milk and shrimp samples. CAP (1 mmol), EGDMA (5 mL), AIBN (120mg) and required amount of 2-(diethylamino)ethyl methacrylate (DEAEM) were dissolved in octanol-chloroform (2:1, *v*/*v*; 15 mL) and added in a flask that contained polyvinyl alcohol 1788 (4 g) dissolved in double distilled water (100 mL). Polymerization was carried out under nitrogen atmosphere and continuous stirring at 70 °C and for 24 h. The developed MIP microspheres were filtered and washed with double distilled water, methanol and acetone and the template was removed by Soxhlet extraction with methanol-acetic acid (90:10, *v*/*v*). The MIP microspheres were washed with methanol and dried under vacuum at 70 °C for 12 h. CAP-MIP microspheres (100 mg) were suspended in isopropanol-methanol (2:1, *v*/*v*; 2 mL) and packed into a SPE cartridge, washed with methanol (5 mL) and preconditioned with phosphate buffer (0.05 M, pH 7.0). The cartridge was loaded with milk sample extract (10 mL), washed with methanol-phosphate buffer (40:60, *v*/*v*; 3 × 1 mL) and the analytes were eluted with methanol (2 × 1 mL). In comparison with the respective NIP, MIP microsphere adsorption capacity for CAP was, with maximum adsorption capacity of 222 μg/g, and higher recoveries for CAP, FFC and TAP [49].

A MISPE-liquid chromatography-mass spectrometry (LC-MS) method was developed for the determination of CAP. CAP-MIPs were prepared by sol-gel synthesis in four steps. In the first step, a self-assembled complex between CAP and the sol-gel precursors was obtained by mixing CAP (250 mg), (3-aminopropyl)triethoxysilane (APTES) (0.8 g), triethoxyphenylsilane (TEPS) (0.8 g) and isopropanol (4 mL). The mixture was sonicated for 30 min and incubated at room temperature for 6 h. In the second step, TMOS (2.5 mL) was mixed with isopropanol (20 mL) and the mixture was vortexed for 5 min. HCl solution (0.1 M, 750 µL) was added to the mixture that was retained in a silicon oil bath at 50 °C for 12 h, until the cross-linker was completely hydrolyzed. In the third step, the mixtures obtained in the first two steps were combined and vortexed for 5 min. The resulting mixture was retained in a silicon oil bath for 4 h, until a transparent gel is formed, and for additional 24 h until the three-dimensional sol-gel network was developed. Finally, the developed MIPs were washed tenfold with methanol (10 mL) with subsequent sonication for 30 min or centrifuging at 1900g for 30 min and dried at 50 °C for 30 min. CAP-MIPs (30 mg) were packed into a syringe barrel, preconditioned with methanol (2 mL) and deionized water (2 mL), retained for 15 min and loaded with defatted and deproteinized spiked milk extract. The analytes were eluted with methanol (500 μL) and the eluate was injected directly for analysis. In comparison with the respective NIP, MIP adsorption capacity for CAP was higher, with maximum adsorption capacity of 2300 μg/g, while imprinting factors were 5 for CAP, 1.9 for TAP and 1.8 for FFC. Furthermore, the MIPs could be used for six consecutive extraction cycles with no adsorption capacity loss [50]. 

A MISPE-high-performance liquid chromatography-diode array detection (HPLC-DAD) method was developed for the determination of CAP in honey and milk samples. Acrylamide-grafted chitosan was used as the matrix in MIP synthesis. Acrylamide (AM) (0.4 g) and CAP (0.1 g) were dissolved in ethyl acetate (10 mL), EGDMA (1 mL) was added and the mixture was stirred for 30 min. 1.5% Chitosan solution (30 mL) dissolved in 2% acetic acid aqueous solution and liquid paraffin (50 mL) that contained sorbitan oleate (0.3 mL) were added and the mixture was stirred at 40 °C for 30 min. The mixture was adjusted to pH 9.0 by adding NaOH solution (2 M), 50% glutaraldehyde (0.8 mL) was added and the mixture was stirred for 3 h. The template was removed by Soxhlet extraction with methanol-acetic acid (9:1, *v*/*v*) for 10 h. CAP-chitosan-MIPs (100 mg) were packed into a SPE cartridge, loaded with deproteinized spiked milk extract (5 mL) and washed with 10% methanol. The analytes were eluted with 10% acetic acid in methanol. In comparison with the respective NIP, MIP adsorption capacity for CAP was higher, with Q_max_ values of 4400 and 59,850 μg/g, while imprinting factors were 7.43 for CAP, 1.29 for erythromycin (ERY) and 1.83 for tetracycline (TC) [51].

A MISPE-LC-MS/MS method was developed for the determination of FFC. FFC (0.358 g) and AM (0.284 g) were dissolved in dimethyl sulfoxide (10 mL) and the mixture was stirred in dark for 1 h. EGDMA (4 mL) and styrene (1 mL) were added and the mixture was stirred for 30 min. The upper phase was collected, mixed with water (50 mL) that contained sodium dodecyl sulfate (150 mg) and stirred for 600 rpm for 25 min. The emulsion was placed inside a high-pressure microwave vessel at 70 °C for 1 h. The template was removed with methanol-acetic acid (80:20, *v*/*v*) and ultrasound application. The MIP particles were washed with water and dried at 60 °C. FFC-MIPs (100 mg) were packed into a syringe barrel, preconditioned with methanol (3 mL) and water (3 mL), loaded with milk extract and washed with acetonitrile-water (50:50, *v*/*v*; 2 mL). The analytes were eluted with 4% acetic acid in methanol (4 mL), the eluate was filtered with a 0.22 μm filter, evaporated under nitrogen stream and the dry residue was reconstituted with acetonitrile-water (50:50, *v*/*v*; 1 mL). In comparison with the respective NIP, MIP adsorption capacity for FFC was higher, with Q_max_ values of 16.2 and 183.1 μmol/g, while binding specificity study between FF, CAP, cefadroxil (CFD) and roxithromycin showed MIP selectivity over FFC and CAP. Furthermore, the use of microwave-assisted emulsion polymerization reaction used in this paper resulted in reduced required time (1 h) and MIP particles with improved morphological characteristics [52].

A MISPE-high-performance liquid chromatography-mass spectrometry (HPLC-MS/MS) method was developed for the determination of CAP, TAP and FFC in baby formula samples. A mixture of CAP (0.19 mmol), MAA (1.5 mmol), DVB (3.84 mmol), AIBN (0.27 mmol) and acetonitrile-toluene (3:1, *v*/*v*; 12.5 mL) was placed inside a temperature controllable incubator at 60 °C and continuous stirring at 24 rpm for 24 h. The developed MIP particles were washed with acetonitrile and methanol and filtered through a 0.45 μm nylon membrane. The template was removed by Soxhlet extraction with methanol-acetic acid (1:1, *v*/*v*) for 8 h. CAP-MIPs (0.05 g) were packed into a SPE cartridge, loaded with formula powder extract (1 mL) and washed with 5% acetonitrile in toluene. The analytes were eluted with 1% acetic acid in methanol, the eluate was evaporated at 30 °C under nitrogen stream and the dry residue was reconstituted in mobile phase. The imprinting effect was studied by comparing the MIPs and the respective NIPs during the sample loading step where a 50% analytes loss was observed with the use of the NIP. Furthermore, the developed method utilized sample deproteinization pre-treatment with ethyl acetate and MISPE clean-up for the extraction and determination of the analytes, while CAP was used as the template molecule for the simultaneous recognition of three structurally related amphenicols [53].

The same laboratory developed two MIPMME-HPLC-DAD methods for the determination of TAP in milk and honey samples. For the first method, TAP (0.05 mmol) was dissolved in toluene (52 mL) that contained dodecanol (0.31 g) and 4-VP (0.1 mmol) and the mixture was strongly sonicated for 5 h. EGDMA (1 mmol) and AIBN (8.5 mg) were added and the mixture was sonicated for 10 min. The mixture (50 mL) was transferred and sealed inside a micropipette tip and polymerization was carried out at 60 °C for 24 h. The developed MIP monoliths were washed by pumping methanol with a syringe infusion pump. The TAP-MIP monolith containing micropipette tip was attached to a syringe infusion pump, preconditioned with methanol (5 mL) and water (2 mL), loaded with pre-treated spiked milk extract (5 g) and washed with water (0.5 mL). The analytes were eluted with methanol (0.1 mL) and the eluate was injected directly for analysis. In comparison with the respective NIP, MIP adsorption capacity for TAP was 183.1 μg/g, while binding specificity study between TAP, CAP, and sulfadiazine (SDZ) showed MIP selectivity over TAP and low affinity for CAP and the imprinting factor was 7.5 for TAP [54]. For the second method, CAP-MIP monoliths were developed by following the same preparation protocol. The developed MIP monoliths were washed with methanol. The TAP-MIP monolith containing micropipette tip was attached to a syringe infusion pump, preconditioned with methanol (2 mL) and water (1 mL), loaded with pre-treated spiked milk extract (4 mL) and washed with water (0.5 mL). The analytes were eluted with methanol-water (55:45, *v*/*v*; 0.1 mL) and the eluate was injected directly for analysis. In comparison with the respective NIP, MIP adsorption capacity for TAP was 62.72 μg/g and the imprinting factor was 2.46 [55]. 

MIP4SPE-CAP cartridges (MIP Technologies, Lund, Sweden) were used for the development of a MISPE-liquid chromatography-electrospray ionization-mass spectrometry (LC-ESI-MS/MS) method for the determination of CAP in raw milk, skimmed milk and milk powder samples. The MIP4SPE-CAP cartridge was preconditioned with methanol (1 mL) and water (1 mL), loaded with pre-treated spiked raw milk extract (1 g) and washed with water (2 mL), water-acetonitrile (95:5, *v*/*v*; 1 mL) that contained 5% acetic acid, water (2 mL) and water-acetonitrile (80:20, *v*/*v*; 1 mL) that contained 1% of a 25% ammonia solution. The cartridge was dried under vacuum for 10 min and washed with dichloromethane (3 mL). The analytes were eluted with water-methanol (10:90, *v*/*v*; 2 mL) that contained 1% acetic acid, the eluate was evaporated under nitrogen stream at 50 °C and the dry residue was reconstituted with deionized water (150 µL). The reconstituted eluate was vortexed, sonicated for 5 min and filtrated through a 0.45 μm filter. Similar sample preparation protocols were developed for the extraction of CAP from skimmed milk and milk powder samples. The authors compared the developed MIP4SPE-CAP protocol with classic SPE protocol with Oasis HLB cartridges (Waters, Rupperswill, Switzerland). The MISPE protocol displayed higher CAP recoveries, lower throughput time and increased selectivity over the SPE protocol [56].

SupelMIP-CAP SPE cartridges (MIP Technologies, Lund, Sweden) were used for the development of a MISPE- HPLC-UV method for the determination of CAP in honey, urine, milk (raw and semi-skimmed) and plasma samples. The SupelMIP-CAP SPE cartridge was preconditioned with methanol (1 mL) and HPLC grade water (1 mL), loaded with spiked raw milk extract (5 mL) and washed with water (2 × 1 mL), acetonitrile-0.5% acetic acid aqueous solution (5:95, *v*/*v*; 1 mL), 1% ammonia aqueous solution (2 × 1 mL) and acetonitrile-1% ammonia aqueous solution (20:80, *v*/*v*, 1 mL). The cartridge was dried under vacuum for 5 min, washed with 2% acetic acid in dichloromethane (2 × 1 mL) and dried for 2 min. The analytes were eluted with methanol-acetic acid-water (89:1:10, *v*/*v*/*v*; 2 × 1 mL), the eluate was evaporated under vacuum at 55 °C and the dry residue was reconstituted with 30% acetonitrile in 10 mM ammonium acetate solution (100 μL) [57]. SupelMIP-CAP SPE cartridges (MIP Technologies, Lund, Sweden) were also used for the development of a MISPE-gas chromatography-negative chemical ionization-mass spectrometry (GC-NCI-MS) method for the determination of CAP in urine, feed water, cow milk and honey samples. The SupelMIP-CAP SPE cartridge was preconditioned with methanol (5 mL) and deionized water (5 mL), loaded with raw cow milk extract (1 mL) and washed with water (2 mL), 5% acetonitrile in 0.5% acetic acid aqueous solution (1 mL), 1% ammonia aqueous solution (2 mL) and 20% acetonitrile in 1% ammonia aqueous solution (1 mL). The cartridge was dried under vacuum for 5 min, washed with dichloromethane (2 mL) and dried for 1 min. The analytes were eluted with dichloromethane-acetic acid-methanol (89:1:10, *v*/*v*/*v*; 2 mL) and the eluate was evaporated to dryness at 40 °C. The SupelMIP-CAP SPE cartridges were compared and found superior to SPE-C18 cartridges (Waters, Milford, MA, USA) in means of sample cleaning capability, simplicity and throughput time [58]. All the reported applications are summarized in Table 1.

### 5.2. Penicillins

Penicillins are the oldest antibiotics class. They belong to the group of β-lactams, along with cephalosporins, and are bactericidal. Penicillins include aminopenicillins such as ampicillin (AMP) and amoxicillin (AMX) that have a broader spectrum of action in comparison with natural penicillins, penicillinase-resistant penicillins such as oxacillin (OXA) and penicillin G (PEN G) structure-based penicillins. The maximum residue limit in milk according to the Commission Regulation (EU) No. 37/2010 is 4 μg/kg for AMP, AMX and benzylpenicillin and 30 μg/kg for OXA, cloxacillin (CLOX), dicloxacillin (DICLOX) and nafcillin (NAFC) [2,3].

MIPs were reported as sorbent materials for the extraction of penicillins from milk samples. Synthesized MIPs were mainly employed in MISPE protocols, along with a report of a MSPE protocol and a report of a MSPD protocol.

Α MISPE-LC-MS/MS method was developed for the determination of AMP in cow milk samples. AMP (0.01 g) was mixed in acetonitrile (10 mL) and the mixture was briefly vortexed at 50 °C. MAA was added and the mixture was vortexed for 1 min and sonicated for 5 min. EGDMA and 2% hydrogen peroxide were added and the mixture was vortexed for 1 min and sonicated for 2 min. The mixture was purged under nitrogen stream for 3 min and polymerization was carried out under UV light lamp at 28 °C for 24 h. The developed MIP bulk was grinded into particles of 300 μm size, filtered and washed with ethanol and methanol. The template was removed by incubating the MIPs in methanol-acetic acid (9:1, *v*/*v*) in a water bath at 50 °C for 6 h. The MIP particles were dried and kept inside a desiccator. AMP-MIPs (350 mg) were packed into a syringe barrel, preconditioned with acetonitrile (5 × 2 mL), loaded with deproteinized and defatted spiked milk extract (20 g) and washed with acetonitrile (2 × 2 mL). The analytes were eluted with methanol-acetic acid (99.4:0.6, *v*/*v*; 3 × 2 mL), the eluate was evaporated under nitrogen stream at 30 °C and the dry residue was reconstituted with water-KH_2_PO_4_-acetic acid (89:10:1, *v*/*v*/*v*; 1 mL). In comparison with the respective NIP, binding specificity study between AMP, AMX, OXA and PEN G showed MIP selectivity over AMP and recoveries were 110% for AMP, 21.8% for AMX, 33.3% for OXA and 15.4% for PEN G [59].

A second MISPE-LC-MS/MS method was reported for the determination of benzylpenicillin. Benzylpenicillin (0.7 g), MAA (1.4 g), TRIM (2.4 g) and AIBN (0.07 g) were dissolved in acetonitrile (70 mL) and the mixture was placed in a water bath, under nitrogen atmosphere at 75 °C for 20 h. The developed MIPs were dried at 60 °C for 48 h and the template was removed by Soxhlet extraction with methanol-water (95:5, *v*/*v*). Benzylpenicillin-MIPs (50 mg) were packed into an empty SPE cartridge, washed with methanol (50 mL) and methanol-water mixture (95:5, *v*/*v*; 15 mL) and preconditioned with methanol-acetonitrile mixture (50:50, *v*/*v*; 5 mL). The cartridge was loaded with pretreated spiked milk extract and washed with acetonitrile (2 mL). The analytes were eluted with methanol-water (50:50, *v*/*v*; 6 mL), the eluate was evaporated under nitrogen stream at 60 °C and the dry residue was reconstituted with water-acetonitrile mixture (1:1, *v*/*v*; 1 mL) and internal standard (1 mL). The reconstituted eluate was filtered through a 0.22 μm filter. In comparison with the respective NIP, MIP adsorption capacity for benzylpenicillin was higher, with Q_max_ values of 0.13 and 0.82 μmol/g, while MIP clean-up recoveries were up to 88% and NIP recoveries (0–11)%. The developed MISPE protocol was compared with a classic sample preparation protocol applied in benzylpenicillin-positive milk samples, showing that MIPSE is a good alternative [60].

A MISPE-HPLC-DAD method was developed for the determination of OXA, CLOX and DICLOX, utilizing 2-biphenylylpenicillin as the surrogate template molecule. 2-B iphenylylpenicillin (0.25 mmol), *N*-(2-aminoethyl)methacrylamide (EAMA) (1 mmol) and 15-crown-5 ether (0.5 mmol) were dissolved in acetonitrile-dimethylsulfoxide (60:40, *v*/*v*; 0.7 mL) and mixed with TRIM (5 mmol) and *N*,*N*′-azo-bis(2,4-dimethyl)valeronitrile.

Silica (6 g) was added to the above mixture (2.5 mL), the mixture was stirred, sealed and purged under nitrogen stream for 5 min and polymerization was carried out at 50 °C for 24 h. The developed MIP particles were treated three consecutive times with ammonium hydrogen difluoride aqueous solution (3 M, 150 mL) for 8 h, washed with water, methanol-trifluoroacetic acid (99:1, *v*/*v*; 1 L), and methanol (0.5 L) and dried under vacuum at 50 °C for 24 h. The MIPs were suspended in methanol-water (80:20, *v*/*v*) in order to remove finer particles. MIPs (20 mg) were packed into a SPE cartridge, equilibrated with methanol (10 mL) and 2-[4-(2-hydroxyethyl)-1-piperazinyl]ethanesulfonic acid buffer (0.1 M, pH 7.5; 10 mL), loaded and rinsed with water-acetonitrile (80:20, *v*/*v*; 3 mL). The analytes were eluted with 0.05 M trifluoroacetic acid in methanol (1 mL) and the eluate (0.4 mL) was diluted with water (0.6 mL). In comparison with the respective NIP, MIP adsorption capacity for CLOX was 124 μmol/g and NIP adsorption capacity was 31 μmol/g, while binding specificity study between OXA, CLOX, DICLOX, AMX, AMP, doxycycline, PEN G and NAFC showed MIP selectivity over OXA, CLOX and DICLOX and imprinting factors were 90.5 for OXA, 80.8 for CLOX and 74.9 for DICLOX [61].

A MISPE-HPLC-MS/MS was developed for the determination of eight penicillin antibiotics, AMP, AMO, OXA, PEN G, penicillin V (PEN V), CLOX, DICLOX and NAFC in baby formula samples. NAF, MAA, EGDMA and AIBN were dissolved in acetonitrile and the mixture was placed inside an incubator at 60 °C and stirring at 24 rpm for 24 h. The developed MIPs were collected and filtered through a 0.45 μm filter using acetonitrile (50 mL) and methanol (50 mL). The template was removed by Soxhlet extraction with methanol-acetic acid (1:1, *v*/*v*) for 8 h. NAFC-MIPs (0.05 g) were packed into an empty SPE cartridge, loaded with pretreated spiked baby formula extract (0.3 g) and washed with acetonitrile (6 × 1 mL). The analytes were eluted with methanol with 1% acetic acid (3 × 1 mL), the eluate was evaporated under nitrogen stream and the dry residue was reconstituted with mobile phase (100 μL) [62].

A magnetic solid-phase extraction (MSPE)-LC-MS/MS method was developed for the determination of PEN V, AMX and OXA. PENV (1 mmol) was dissolved in water-ethanol (9:1, *v*/*v*; 10 mL) and MAA (8 mmol) by stirring for 30 min and EGDMA (20 mmol) were added. The mixture was mixed with Fe_3_O_4_ particles (1 g) and oleic acid (1 mL) and sonicated for 30 min in order to form a pre-polymerization mixture. Polyvinylpyrrolidone (0.4 g) was dissolved in methanol (100 mL) inside a three-necked round-bottomed flask, the mixture was purged under nitrogen stream, stirred at 300 rpm and heated at 60 °C. Then, the pre-polymerization solution and AIBN (1 g) were added into the flask and polymerization was carried out at 60 °C for 24 h. The developed MMIPs were collected with a magnet, washed with methanol-acetic acid (8:2, *v*/*v*) and the template was removed by washing the MMIPs with methanol. The MMIPs were washed three times with water and dried at 60 °C. PENV-MMIPs (100 mg) were preconditioned with methanol (3 mL) and water (3 mL), dispersed in a flask that contained milk sample (2 mL) and hydrochloric acid aqueous solution (pH 5.0, 18 mL) and sonicated for 5 min. The MMIPs were then collected and washed with water (3 mL) and the analytes were eluted with 5% acetic acid in methanol (3 × 1 mL) and sonication for 30 s. The eluate was evaporated at 40 °C, the dry residue was reconstituted with 1% formic acid in methanol (1 mL) and filtered through a 0.45 μm membrane. In comparison with the respective NIP, MIP adsorption capacity for PEN V was higher, with Q_max_ values of 70.71 and 139.88 μmol/g. Furthermore, the proposed extraction protocol was effortless and less time consuming in comparison with a conventional SPE protocol, because it provides easier and faster sample loading, sorbent collection and washing and analyte elution [63].

A MSPD-HPLC-UV was developed for the determination of penicillin degradation products, penicilloic acid and penilloic acid. Penicilloic acid (0.5 mmol) was chosen as the template molecule, MAA (2 mmol) as the functional monomer, EGDMA (5 mmol) as the cross-linker, methanol-acetonitrile (1:1, *v*/*v*; 20 mL) as the solvent, AIBN (16.4 mg) as the initiator and modified silica gel (20 mg) as the support. Polymerization was carried out at 60 °C for 24 h and the template was removed by Soxhlet extraction. Penilloic acid-selective surface molecularly imprinted polymers (SMIPs) and spiked milk sample were mixed at a ratio of 3:2 into a glass mortar, blended with a glass pestle and the dry homogenous mixture was packed into an empty cartridge. The cartridge was washed with dichloromethane (2 mL) and the analytes were eluted with 10% acetic acid in methanol (3 mL). The eluate was evaporated under nitrogen stream at room temperature, the dry residue was reconstituted with mobile phase (3 mL) and the reconstituted eluate was filtered through a 0.45 μm filter. In comparison with the respective NIP, MIP adsorption capacity for penicilloic acid and penilloic acid was higher, with Q_max_ values of 22,670 and 21,090 μg/g respectively, while binding specificity study between penicilloic acid, penilloic acid, CLOX, AMP and OXA showed SMIP selectivity over penicilloic acid, penilloic acid and imprinting factors were 6.3 for penicilloic acid and 3.5 for penilloic acid. Furthermore, the authors compared the developed MSPD protocol with three commercially available SPE cartridges, Cleanert PAX (500 mg, 6 mL), Cleanert PCX (500 mg, 6 mL) and Cleanert Florisil (500 mg, 6 mL) purchased from Bonna-Agela Technologies Inc. (Newark, USA), in terms of selectivity and extraction efficiency. The MSPD protocol displayed highest recoveries than the SPE protocols, with 88.5% recovery for penicilloic acid and 85.9% for penilloic acid [64]. All the reported applications are summarized in Table 2.

### 5.3. Quinolones

Quinolones constitute a broad-spectrum antibiotic class that includes OXO. Quinolones inhibit the bacterial DNA synthesis and the first quinolones were used for urinary tract infection treatment. More effective fluoroquinolones were synthesized by adding a fluorine atom in the basic heterocyclic aromatic structure of the quinolones. The maximum residue limit in milk according to the Commission Regulation (EU) No. 37/2010 is 100 μg/kg for the sum of enrofloxacin (ENR) and ciprofloxacin (CIP), 30 μg/kg for danofloxacin (DAN), while difloxacin (DIF) and FFC are not recommended for milk producing animals [1,2,3].

Multi-template MIPs used in MISPE protocols, a MIP column used in on-line MISPE, a PMME protocol, along with a DSPE protocol and a MSPE protocol were reported in the literature for the extraction of various quinolones from milk samples.

A MISPE-HPLC-DAD method was developed for the determination of 10 quinolone antibiotics: fleroxacin (FLX), enoxacin (ENO), pefloxacin (PEF), norfloxacin (NOR), CIP, levofloxacin (LVFX), lomefloxacin (LOM), ENR, gatifloxacin (GAT) and sparfloxacin (SPA), utilizing multiple-template surface molecularly imprinted polymers (MTMIPs). CIP (165.5 mg), LVFX (180.6 mg) MAA (517.4 mg) were dissolved in toluene (6 mL) and the mixture was sonicated. TRIM (5.408 g) and AIBN (56 mg) were added and the mixture was sonicated, degassed under nitrogen stream for 10 min, sealed and placed in a water bath 60 °C for 24 h. The MIP bulk was grinded into particles of 54–74 µm diameter and the template was removed by incubating the MIPs in methanol-acetic acid (8:2, *v*/*v*) under sonication followed by centrifuging at 6000 rpm. The MIP particles were washed with methanol until pH 7.0 and dried. MTMIPs (200 mg) were packed into a SPE cartridge, preconditioned with methanol (3 mL) and water (3 mL), loaded with pretreated spiked milk extract (1 mL) and washed with water (3 mL). The analytes were eluted with 4% ammonia in methanol (3 mL), the eluate was evaporated under nitrogen stream at 45 °C, the dry residue was reconstituted with mobile phase (1 mL) and filtered through a 0.22 μm filter. In comparison with the respective NIP, MIP adsorption capacity for CIP acid LVFX was higher, with Q_max_ values of 32.3 and 94.2 μmol/g for CIP and 32.5 and 71.9 μmol/g for LVFX. The developed MIPs could be successfully applied for the extraction and determination of 10 fluoroquinolone antibiotics by utilizing two template molecules rather than one and were reused for 20 extraction cycles with no extraction efficiency reduction [65].

MTMIPs were also utilized for the development of a MISPE-HPLC-UV method for the determination of estriol, estrone, 17β-estradiol, ofloxacin (OFL), NOR and CIP. OFL (0.1 mmol) and 17β-estradiol (0.1 mmol) were dissolved in acetonitrile (15 mL), sufficient amount of MAA was added and the mixture was stirred at 50 rpm. Modified monodispersed P_GMA/EDMA_ beads (0.8 g), EGDMA (3 mmol) and AIBN (0.1 g) were added and the mixture was transferred in a water bath at 65 °C under nitrogen atmosphere and continuous stirring at 120 rpm for 24 h.

The P_GMA/EDMA_ beads acted as support for the MIPs and were previously prepared by dispersion polymerization and chemically modified according to previous work. The developed MIPs were washed with water and methanol and the templates was removed by incubating the MIPs in acetic acid-methanol (80:20, *v*/*v*; 50 mL) under continuous stirring for 24 h. The MIP particles were washed with distilled water and air-dried. MTMIPs (50 mg) were packed into a polypropylene cartridge, loaded with pretreated spiked milk extract (5 mL) and washed with water-methanol (9:1, *v*/*v*; 1 mL). The analytes were eluted with 20% acetic acid in acetonitrile (3 × 0.5 mL), the eluate was evaporated under nitrogen stream at 25 °C and the dry residue was reconstituted with of mobile phase (20 μL). In comparison with the respective NIP, MIP adsorption capacity for OFL and 17β-estradiol was higher, with maximum adsorption capacity of 9000 and 6600 μg/g respectively, while binding specificity study between OFL, 17β-estradiol, NOR, estriol and CAP showed SMIP selectivity over OFL and 17β-estradiol and imprinting factors were 6.7 for OFL, 12 for 17β-estradiol, 4.8 for NOR, 2.8 for estriol and 1.1 for CAP. The chromatograms demonstrated better peaks with no matrix interference for the MISPE protocol in contrast with the C18 SPE [66].

An on-line MISPE-HPLC-UV method was developed for the determination of OFL, LOM, CIP and ENR, utilizing a molecularly imprinted polymer hybrid composite monolithic column (MIP-HCMC). ENR (0.18 g) dissolved in toluene (0.5 mL) and ethanol (0.5 mL) was mixed with MAA (0.1 mL) and HEMA (0.15 mL) and the mixture was stirred at 52 °C under nitrogen stream for 1 h. TEOS (2.32 mL) dissolved in acetonitrile (3.55 mL) and HCl (0.10 mL) were added and the mixture was incubated for 1 h. 3-methacryloxypropyltrimethoxysilane (KH-570) (0.58 mL), EGDMA (0.25 mL) and AIBN (0.06 g) were added, the mixture was sonicated for 2 min, transferred and sealed inside a stainless-steel column and polymerization was carried out at 53 °C for 12 h. The template was removed with methanol-acetic acid (4:1, *v*/*v*) and methanol and the column was connected to an on-line SPE-HPLC system. The MIP column preconditioned with water-acetonitrile (75:25, *v*/*v*; 2 mL) and water-acetonitrile (90:10, *v*/*v*; 2 mL), loaded with pretreated spiked milk extract, washed with methanol-water (7:93, *v*/*v*; 1.5 mL) and the analytes were eluted with mobile phase. MIP dynamic adsorption capacity was 4.08 mg/g for ENR, while NIP adsorption capacity was and 2.44 mg/g [67].

A PMME-high-performance liquid chromatography-fluorescence detection (HPLC-FLD) method was developed for the extraction and determination of CIP, DIF, DAN and ENR. PEF (24 mg), MAA (40 mg), di(ethylene glycol) dimethacrylate (DEGDMA) (240 mg) and AIBN (5 mg) were dissolved in methanol-water (10:3, *v*/*v*; 0.8 mL) and the mixture was sonicated for 10 min. The mixture was transferred and sealed inside activated and modified fused-silica capillaries and polymerization was carried out at 65 °C for 16 h. The developed MIP monoliths were washed with methanol-trifluoroacetic acid (98:2, *v*/*v*) for 48 h by connecting the capillary columns with a HPLC pump. The needle of a syringe pinhead was replaced with a PEF-MIP monolith capillary and all the extraction steps (precondition, loading, washing and elution) were employed by means of a syringe infusion pump. The PEF-MIP monolith was preconditioned with 2% trifluoroacetic acid in methanol (2 mL) and phosphate buffer solution (25 mM, pH 6.0; 0.4 mL), loaded with pretreated spiked milk extract (1 mL) and washed with acetonitrile-phosphate buffer (15:85, *v*/*v*; 0.2 mL). The analytes were eluted with 2% trifluoroacetic acid in methanol (0.4 mL), the eluent was evaporated under mild nitrogen stream at 50 °C and the dry residue was reconstituted with 100 μL of mobile phase. In comparison with the respective NIP, MIP adsorption capacity for PEF was higher, with maximum adsorption capacity of 36 μmol/g, while imprinting factors were 3.1 for PEF and 1 for FLU. Binding specificity study between fluoroquinolones (PEF, CIP, DAN, ENR, DIF) and quinolones (FLU, OXO) showed MIP selectivity over the fluoroquinolones, with recoveries up to 81.6% for the fluoroquinolones and less than 17.6% for the quinolones. Furthermore, the developed PMME protocol was compared with classic C18 SPE protocol. The chromatograms demonstrated no matrix interference for the PMME protocol in contrast with the C18 SPE protocol, proving once more the selectivity of the developed method [68].

A DSPE-HPLC-UV method was developed for the determination of OFL, NOR and ENR. SiO_2_ nanoparticles (1 g) and 0.5 mL of KH-570 were dispersed in ethanol, the mixture was stirred 25 °C for 24 h and the SiO_2_-KH-570 particles were dried under vacuum. OFL (0.1 mmol), MAA (0.8 mmol), and SiO_2_-KH-570 particles (0.5 g) were dispersed in acetonitrile (20 mL) and the mixture was placed in an ice bath and stirred for 8 h. EGDMA (4 mmol), AIBN (0.12 mmol) and dibutyl isophthalate (0.28 mmol) were added and polymerization was carried out under nitrogen stream and stirring at 60 °C for 24 h. The developed MIPs (100 mg) were treated with 40% HF aqueous solution, vortexed for 15 min, kept for 2 h, centrifuged, washed with methanol and dried under vacuum. Hollow OFL-MIPs (20 mg) were dispersed in pretreated spiked milk extract (10 mL) and the mixture was shaken for 15 min and centrifuged. The analytes were eluted, the eluate was evaporated and the dry residue was reconstituted with mobile phase. In comparison with the respective NIP, MIP adsorption capacity for OFL was higher, with Q_max_ values of 133 and 274 μmol/g, while binding specificity study between OFL, sulfamethazine (SMZ), ibuprofen and Sudan I showed MIP selectivity over OFL and imprinting factor was 2.6 for OFL [69].

A MSPE-HPLC-UV method was developed for the extraction and determination of OFL, CIP and LOM. SupelMIP-fluoroquinolones SPE (Sigma-Aldrich, St. Louis, MO, USA) sorbent material (20 mg) and Fe_3_O_4_ magnetite nanoparticles (20 mg) were mixed with methanol (1 mL) and the mixture was strongly vortexed for 1 min. The MMIPs were collected by means of an external magnet, the supernatant was discarded, ultra-pure water (2 mL) was added to the MIPs, the mixture was vortexed for 1 min and the supernatant was discarded. Pretreated spiked milk extract (2 mL) was added and the mixture was vortexed for 1 min. The MMIPs were collected with a magnet, washed with ultra-pure water (3 mL), acetonitrile (1 mL) and 15% acetonitrile in ultra-pure water (1 mL). The analytes were eluted with 7% ammonia in methanol (1 mL) and vortexing for 2 min, the MMIPs were removed by means of a magnet, the eluate was evaporated under nitrogen stream at 35 °C and the dry residue was reconstituted with mobile phase. The developed MSPE protocol was compared with a MISPE protocol and showed similar or even increased clean-up efficiency and higher preconcentration capability for the examined quinolones [70]. All the reported applications are summarized in Table 3.

### 5.4. Tetracyclines

Tetracyclines are the first broad-spectrum antibiotic class that was discovered in 1945 from *Streptomyces* bacteria. They inhibit the bacterial protein synthesis and first tetracyclines to be introduced for clinical use were chlortetracycline and tetracycline. This group of antibiotics is characterized by a four-ring basic structure and can be effective against a variety of microorganisms, such as rickettsia and amoebic parasites. The maximum residue limit in milk according to the Commission Regulation (EU) No. 37/2010 is 100 μg/kg for chlortetracycline (CTC), oxytetracycline (OTC) and TC, while doxycycline (DC) is not recommended for milk producing animals [1,2,3].

MIPs were reported as sorbent materials for the extraction of tetracycline antibiotics from milk samples. Synthesized MIPs were mainly employed in MISPE protocols with reports of a PMME, a SPME and two magnetic dispersion extraction (MDE) protocols.

A MISPE-HPLC-DAD method was developed for determination of CTC, TC, OTC and DC in milk, egg and pork samples. CTC and MAA were dissolved in chloroform (7 mL) and the mixture was shaken for 1 min, sonicated for 15 min and stored at 4 °C overnight. EGDMA (0.3 mmol) and AIBN (40 mg) were added, the mixture was degassed under nitrogen stream for 10 min, sealed and placed in a water bath at 60 °C for 24 h. The developed MIP bulk was crushed into particles of (32–60) µm size, the template was removed by Soxhlet extraction with methanol-acetic acid (8:2, *v*/*v*) for 48 h, and the MIP particles were dried at 110 °C. CTC-MIPs (30 mg) were packed into an empty cartridge, preconditioned with ultrapure water (3 mL) and methanol (3 mL) and loaded with pretreated spiked milk extract. The loaded cartridge was washed with ultrapure water (3 mL) and methanol (3 mL) and dried under vacuum for 30 s. The analytes were eluted with methanol-acetic acid (9:1, *v*/*v*; 1 mL) and the eluate was filtered through a 0.22 µm nylon filter.

Binding specificity study between CTC, TC, OTC, DC, sulfadimidine, CIP and AMX showed MIP selectivity over the studied tetracyclines, with recoveries higher than 87% for CTC, TC, OTC and DC, between (20–27)% for sulfadimidine, CIP and AMX, while NIP recoveries were between (20–23)% for all compounds. The developed MISPE protocol was compared with classic SPE protocols utilizing three commercially available SPE cartridges. The chromatograms demonstrated no matrix interference for the MISPE protocol in contrast with the SPE protocols where interfering peaks were observed. Furthermore, the developed MISPE cartridges could be reused for 30 extraction cycles with no extraction efficiency reduction [71].

The same laboratory developed three MISPE-HPLC-UV and an on-line MISPE-HPLC-UV method, utilizing molecularly imprinted polymer hybrid composite materials (MIP-HCMs) for the extraction of various tetracycline antibiotics.

A MISPE-HPLC-UV method was developed for the determination of TC, CTC and DC, utilizing a hydrophilic MIP-HCM. DC (0.151 g), MAA (0.22 mL), DVB (0.2 mL) and styrene (0.6 mL) were dissolved in acetonitrile (2.25 mL) and the mixture was left to pre-polymerize for 30 min. KH-570 (1.176 mL) and AIBN (0.1 g) were added, the mixture was degassed by sonication for 2 min, purged under nitrogen stream and heated to 60 °C for 1 h and polymerization was carried out at 60 °C for 3 h. TEOS (2.95 mL) dissolved in HCl (0.23 mL) and ethanol (4.525 mL) was added and mixture was stirred 60 °C for 3 h. The developed MIP-HCM particles were filtered, dried 60 °C for 24 h and the template was removed by Soxhlet extraction with methanol-acetic acid (4:1, *v*/*v*) for 4 h. The MIP particles were treated with KH-570, washed with toluene (100 mL) and acetone (100 mL) and dried at 40 °C overnight. DC-MIP-HCMs (50 mg) were packed into a SPE cartridge, preconditioned with methanol (3 mL) and water (3 mL), loaded with pretreated spiked milk extract (5 mL), washed with methanol-water (20:80, *v*/*v*; 2 mL). The analytes were eluted with methanol-acetic acid (60:40, *v*/*v*; 2 mL), the eluate was evaporated under nitrogen stream and the dry residue was reconstituted with mobile phase. The developed MISPE protocol demonstrated good clean-up efficiency and high selectivity over TC, CTC and DC [72].

A MISPE-HPLC-UV method was developed for the determination of DC, TC and CTC in milk and honey samples. DC (0.151 g) and MAA (0.220 mL) were dissolved in acetone (4.588 mL) and the mixture was incubated at room temperature for 1 h. KH-570 (0.588 mL) and AIBN (75 mg) were added and the mixture was sonicated for 2 min, degassed under nitrogen stream for 5 min and stirred at 53.8 °C for 3 h. TEOS (2.731 mL) dissolved in ethanol (6 mL) and hydrolyzed with HCl (0.1 mL) was added and the mixture was left to react at 60 °C under continuous stirring for 3 h. The mixture was cooled, adjusted to pH 6.0 with ammonia solution and polymerization was carried out by heating at 80 °C for 3 h. The developed MIP-HCM was grinded into particles of (38.5–63) μm size and the template was removed by Soxhlet extraction with methanol-acetic acid (4:1, *v*/*v*) for 24 h. The MIP-HCM particles were washed with methanol and dried at 120 °C for 24 h. DC-MIP-HCM particles (50 mg) were packed into an empty SPE cartridge, preconditioned with water-acetic acid (90:10, *v*/*v*; 3 mL) and water (4 mL), dried for 2 min, further washed with cyclohexane (1 mL) and dried for 2 min. The MISPE cartridge was loaded with pretreated spiked milk extract (4 mL), dried for 3 min and washed with toluene-methanol (85:15, *v*/*v*; 1 mL) and the analytes were eluted with methanol-acetic acid (60:40, *v*/*v*; 3 mL). The developed MIP-HCMs demonstrated higher adsorption capacity and selectivity than the corresponding NIP-HCMs. Furthermore, the MIP-HCMs displayed great rebinding ability by selectively interacting and trapping the analytes in the imprinted sites [73].

A MISPE-HPLC-UV method was developed for the determination of OTC, metacycline (MTC) and DC. OTC (0.160 g) and MAA (0.207 mL) were dissolved in acetonitrile (1.125 mL) and the mixture was incubated at room temperature for 1 h. KH-570 (0.554 mL) and AIBN (0.068 g) were added and the mixture was sonicated for 2 min and stirred under nitrogen stream at 53.8 °C for 3 h. TEOS (2.73 mL) dissolved in ethanol (5.60 mL) and hydrolyzed with HCl (0.1 mL) was added and the mixture was stirred at 60 °C for 3 h. The mixture was adjusted to pH 6.0 with sodium hydroxide solution and evaporated at 65 °C. The developed MIP-HCM was grinded into particles of (38.5–63) μm size and the template was removed by Soxhlet extraction with methanol-acetic acid (4:1, *v*/*v*) for 24 h. The MIP-HCM particles were washed with methanol and dried under vacuum at 60 °C for 24 h. OTC-MIP-HCM particles (50 mg) were packed into an empty SPE cartridge, preconditioned with water-acetic acid (90:10, *v*/*v*; 3 mL), water (3 mL), methanol (3 mL) and cyclohexane (3 mL), loaded with pretreated spiked milk extract (4 mL) and washed with toluene-methanol (85:15, *v*/*v*; 1 mL). The analytes were eluted with methanol-acetic acid (60:40, *v*/*v*; 2 mL), the eluate was evaporated under nitrogen stream and the dry residue was reconstituted with mobile phase. In comparison with the respective NIP, MIP adsorption capacity for OTC was higher, with Q_max_ values of 46,650 and 64,800 μg/g [74]. The same MIP-HCM preparation protocol was followed for the preparation of TC-MIP-HCM particles, utilized for the development of a MISPE-HPLC-UV method for the determination of TC, OTC and demeclocycline (DMC) in egg, milk, and milk powder. A pre-column was packed with TC-MIP-HCM particles and connected to an on-line SPE-HPLC system. The pre-column was conditioned with water (2 mL), loaded with pretreated milk extract and washed with methanol-water (5:95, *v*/*v*). The analytes were eluted with methanol-acetonitrile-oxalic acid (5:25:70, *v*/*v*/*v*). Dynamic adsorption capacity for TC was 1790 μg/g [75].

A PMME-HPLC-UV method was developed for the determination of TC, OTC, CTC and minocycline (MC), utilizing MC-imprinted poly(methacrylic acid-KH-570-graphene oxide-*N*,*N*′-methylenebisacrylamide) monoliths. MAA (15 μL), *N*,*N*′-methylenebisacrylamide (MBA) (0.03 g), AIBN (5 mg), KH-570-graphene oxide nanosheets (0.005 g) were dissolved in dimethylsulfoxide (375 mL) with polyethylene glycol (0.11 g) and the mixture was degassed by sonication for 30 min. The mixture was transferred and sealed inside a pretreated capillary and polymerization was carried out at 55 °C for 24 h. The capillary was washed with methanol, MC was pumped through the capillary for 2 h and the excess of MC was removed by pumping tris(hydroxymethyl)aminomethane-HCl buffer (10 mM, pH 8.0) through the monolith. Tris(hydroxymethyl)aminomethane-HCl buffer (10 mM, pH 8.0; 1 mL) that contained dopamine and ammonium persulphate was pumped though the capillary for 15 min so that a polydopamine film was attached on the surface of the developed MIP monolith. The template was removed by washing the monolith with oxalic acid-methanol-acetonitrile (70:10:20, *v*/*v*/*v*). The developed MC imprinted monolith was preconditioned with methanol for 10 min, loaded with pretreated spiked milk extract for 25 min and the analytes were eluted with oxalic acid-methanol-acetonitrile (70:10:20, *v*/*v*/*v*). In comparison with the respective NIP, MIP adsorption capacities for TC, OTC, CTC and MC ranged between (21.28–40.43) μg/g, while the imprinting factors ranged between (1.37–1.89) [76]. 

A SPME-HPLC-FLD method was developed for the determination of OTC, TC, DC and CTC in chicken feed, chicken muscle and milk samples. TC (55.6 mg) and AM (71.1 mg) were dissolved in acetone (11.2 mL) and the mixture was stirred for 12 h. TRIM (1.24 mL) and AIBN (12.4 mg) were added and the mixture (1.5 mL) was transferred into glass tube and degassed under nitrogen stream for 5 min. Etched and silylated fibers were immersed and sealed inside the glass tubes and polymerization was carried out inside a nitrogen evaporator at 60 °C for 6 h. The coated fibers were collected, placed in tubes filled with nitrogen and heated at 60 °C for 24 h. The coating procedure was repeated until desired average thickness of 19.5 μm. The template was removed by soaking the developed TC-MIP coated fibers in methanol-acetic acid (90:10, *v*/*v*; 5 mL) for 30 min. TC-MIP coated fibers were applied to pretreated spiked milk extracts in toluene, with an extraction time of 30 min and stirring at 750 rpm and the analytes were eluted with methanol-buffer (30:70, *v*/*v*). In comparison with the respective NIP, binding specificity study between OTC, TC, DC, CTC, phenol and propranolol showed MIP selectivity over OTC, TC, DC and CTC. Furthermore, the developed fibers could be reused for more than 100 extraction cycles [77].

The same laboratory developed two MDE-HPLC-UV methods for the determination of OTC, TC, CTC and DC, utilizing DC- molecularly imprinted magnetic microspheres (MIMMs) and CTC-MIMMs respectively. In the first report, AIBN (0.1 g) and nonionic surfactant (5 mL) were mixed by stirring until full dispersion. DC (0.48 g), MAA (0.9 mL), AM (0.36 g) and water (2 mL) were added and the mixture was strongly stirred and purged under nitrogen stream for 30 min. Fe_3_O_4_ particles (1 g) were added and the mixture was stirred for 10 min. TRIM (10 mL) and AIBN (0.1 g) were added and the mixture was sonicated until full dispersion. Hydroxyethyl cellulose (0.12 g) dissolved in water (100 mL) was added and the mixture was purged under nitrogen stream for 10 min and polymerization was carried out at 70 °C under continuous stirring for 12 h. The developed MIMMs were collected and washed with water, acetone, ethanol and water. DC-MIMMs (50 mg) were preconditioned with methanol (3 mL) and water (3 mL) and dispersed in pretreated milk sample (4 g). The MIMMs were collected by means of a magnet and washed with methanol-toluene (1:19, *v*/*v*; 2 mL), the analytes were eluted with 0.5% acetic acid in methanol (2 mL), the eluate was evaporated under nitrogen stream at 40 °C and the dry residue was reconstituted with mobile phase. In comparison with the respective NIP, MIP adsorption capacity for OTC, TC, CTC and DC were higher [78]. The same MIMM preparation and sample preparation protocol were followed for preparation of CTC-MIMMs utilized for the extraction of OTC, TC, CTC and DC. In comparison with the respective NIP, MIP adsorption capacity for CTC was higher, with Q_max_ values of 10,690 and 71,460 μg/g [79]. All the reported applications are summarized in Table 4.

### 5.5. Cephalosporins, Macrolides and Sulfonamides

Cephalosporins are grouped into first, second, third, and fourth generations, with broader spectrum in each generation and closely related to cephamycins and carbapenems. They inhibit the bacterial cell synthesis. The maximum residue limit in milk according to the Commission Regulation (EU) No. 37/2010 is 100 μg/kg for cephalexin (CFL), 60 μg/kg for cephapirin (CFP), 50 μg/kg for cefazolin (CFZ) and 20 μg/kg for QUI [1,2,3].

MIPs were only reported as sorbent materials in three MISPE protocols for the extraction of cephalosporin antibiotics from milk samples.

A MISPE-HPLC-UV method was developed for the determination of CFL. CFL (365.4 mg), TFMAA (560.2 mg) and EGDMA (3.9644 g) were dissolved in methanol (5 mL), the mixture was vortexed and sonicated for 5 min and purged under nitrogen stream. AIBN (1.5 mL) was added and the mixture was placed and sealed inside a glass ampule. The glass ampule was placed in a water bath at 60 °C for 14 h. The developed MIPs bulk was grinded and the template was removed with thermal annealing at 100 °C for 18 h, Soxhlet extraction with water-acetic acid (80:20, *v*/*v*) and methanol in an orbital shaker. The MIPs were washed with distilled water in order to remove methanol and dried under vacuum. CFX-MIPs (1 g) were packed into a SPE cartridge, washed with water to remove air, preconditioned with methanol (12 mL) and water (12 mL), loaded with defatted and deproteinized spiked milk extract (5 mL) and washed with methanol (12 mL). The analytes were eluted with water (12 mL), the eluate was lyophilized and the dry residue was reconstituted in methanol-acetate buffer (40:60, *v*/*v*; 500 μL).

In comparison with the respective NIP, MIP adsorption capacity for CFL ranged between (2080–3620) μg/g, while binding specificity study between CFL, ceftazidime, AMP and TC showed MIP selectivity over CFL. Furthermore, the developed MISPE cartridges were compared with commercially available C18 SPE cartridges and were found superior in terms of CFX selectivity and matrix interferences removal. Additional experiments showed that the synthesized MIPs were stable for 12 months without any CFX binding loss [80].

A MISPE-HPLC-UV method was developed for the determination of CFL and CFP, utilizing tributylammonium CFD salt as the template molecule. Tributylammonium CFD (173 mg), MAA (135 μL), and EGDMA (1520 μL) were dissolved in acetonitrile (2.20 mL) and methanol (200 μL) and the mixture was sonicated. AIBN (28.5 mg) was added, the mixture was purged under nitrogen stream for 10 min, sealed and transferred in a water bath 60 °C for 20 h. The developed MIP monoliths were grinded and sieved. The MIP particles were washed with water-acetic acid (4:1, *v*/*v*) and methanol, finer particles were separated by sedimentations in acetone and the MIP particles were dried under vacuum. Tributylammonium CFD-MIPs (100 mg) were packed into a SPE cartridge, preconditioned with phosphoric acid (1.2 mM, 7.5 mL), loaded with deproteinized spiked milk extract (2 mL) and washed with acetonitrile (1 mL). 

The analytes were eluted with methanol-acetic acid (90:10, *v*/*v*; 4 mL), the eluate was evaporated under nitrogen stream at 20 °C and the dry residue was reconstituted with mobile phase. In comparison with the respective NIP, MIP adsorption capacity for CFL was higher, while imprinting factors were 3 for CFD, 5.6 for CFP and 3.8 for CFL [81].

A MISPE-UHPLC-MS/MS method was developed for the determination of cefthiofur (THIO), CFZ, cephalonium (ALO), CFP, cefquinome (QUI) and CFL, utilizing sodium 7-(2-biphenylylcarboxamido)-3-methyl-3-cepheme-4-carboxylate as the surrogate template molecule. Template molecule (0.24 mmol), 15-crown-5 ether (0.24 mmol) and *N*-3,5-bis(trifluoromethyl)phenyl-*N*′-4-vinylphenyl urea (VPU) (0.48 mmol) were dissolved in acetonitrile-dimethylsulfoxide (0.8:1, *v*/*v*; 550 μL) and sufficient amount of DVB and 2,2′-azobis(2,4-dimethylvaleronitrile) was added. The mixture was mixed with silica beads (5 g) by stirring inside a glass vial. The vial was sealed and purged under nitrogen stream for 5 min and polymerization was carried out at 60 °C for 24 h. The developed particles were shaken with ammonium hydrogen difluoride aqueous solution (3 M, 3 × 140 mL) for 24 h, washed with water, methanol-trifluoroacetic acid (99:1, *v*/*v*; 1 L) and methanol (0.5 L) and dried under vacuum at 60 °C for 24 h. The MIP particles were suspended in methanol-water (80:20, *v*/*v*) in order to remove finer particles. MIPs (20 mg) were packed into a SPE cartridge preconditioned with methanol (10 mL) and phosphate buffer (0.05 M, pH 7.5; 10 mL), loaded with pretreated milk extract (10 mL) and washed with HEPES buffer (2:98, *v*/*v*; 5 mL). The analytes were eluted with 0.1% trifluoroacetic acid in methanol (1 mL) and the eluate was diluted with water (800 μL). In comparison with the respective NIP, MIP extraction recoveries for the studied cephalosporins ranged between (74–95)%, while NIP recoveries ranged between (0–8)% for CFZ, ALO, CFP, QUI and CFL [82].

Macrolides constitute a medium-spectrum semi-synthetic antibiotic class, with a macrocyclic lactone chemical structure, discovered in 1950. Macrolides inhibit the bacterial protein synthesis. ERY is the first macrolide antibiotic, with similar spectrum and uses to penicillin, administered as an alternative to people allergic to penicillin. Newer macrolide antibiotics include azithromycin and clarithromycin, which is used stomach ulcer treatment. The maximum residue limit in milk according to the Commission Regulation (EU) No. 37/2010 is 20 μg/kg for ERY and 200 μg/kg for spiramycin (SPI) [1,2,3].

Only two MISPE applications for the extraction of macrolides from sheep milk samples were reported in the literature. The same laboratory developed two MISPE-HPLC-DAD methods were reported for the determination of SPI and ERY in sheep milk samples. For the first method, SPI (0.02 mmol) and MAA (2 mmol) were dissolved in acetonitrile (7 mL) and the mixture was sonicated for 5 min. EGDMA (10 mmol) and AIBN (5.1 mmol) were added and the mixture was sonicated for 10 min, degassed under nitrogen stream for 7 min and placed in a water bath at 50 °C for 4 h. The developed MIP bulk was grinded into particles of (200–355) μm size and the template was removed by Soxhlet extraction with methanol (80 mL) for 20 h. SPI-MIPs (200 mg) were packed into a syringe barrel, preconditioned with methanol (3 × 2 mL) and acetonitrile (3 × 2 mL), loaded with deproteinized spiked milk extract (1 mL) and washed with acetonitrile (3 × 2 mL). The analytes were eluted with acetic acid in methanol (3 × 2 mL), the eluate was evaporated under nitrogen stream and the dry residue was reconstituted with 1 mL of NaH_2_PO_4_-acetonitrile (7:3, *v*/*v*; 1 mL). In comparison with the respective NIP, MIP adsorption capacity for SPI was higher, with Q_max_ values of 1270 and 3560 μg/g, while binding specificity study between SPI, ERY, josamycin, ivermectin and tylosin hemitartrate showed MIP selectivity over SPI and the imprinting factor was 3.4 for SPI [83]. For the second method, the same MIP preparation protocol was followed for the preparation of ERY-MIPs. ERY-MIPs (200 mg) were packed into an empty SPE cartridge, preconditioned with methanol (3 × 2 mL) and acetonitrile (3 × 2 mL) and loaded with pretreated spiked milk extract (1 mL). The loaded cartridge was treated with hexane (6 × 1 mL), in order to remove the milk fat. The analytes were eluted with 0.5% acetic acid in methanol (3 × 2 mL), the eluate was evaporated under gentle nitrogen stream at 40 °C and the dry residue was reconstituted with NaH_2_PO_4_-acetonitrile (7:3, *v*/*v*; 1 mL). In comparison with the respective NIP, binding specificity study between ERY, SPI, roxithromycin, josamycin, ivermectin and tylosin hemitartrate showed MIP selectivity over ERY with recoveries higher than 98% for ERY and between (15–35)% SPI, josamycin, ivermectin and tylosin hemitartrate. The developed MIPs were applied for more than 200 extraction rounds without any performance and stability drop [84].

Sulfonamides constitute synthetic antibiotic class that inhibit the bacterial DNA synthesis and include sulfamethoxazole (SMO), SDZ and SMZ. They contain the sulphonamide functional group (–S(=O)_2_-NH_2_) and are used for bacterial and fungal infection treatment. The maximum residue limit in milk according to the Commission Regulation (EU) No. 37/2010 is 100 μg/kg for all sulfonamide analogues [2,3].

MIPs were reported as sorbent materials in a MISPE, a MSPE, a PMME and a SBSE protocol for the extraction of sulfonamides from milk and other matrices.

A MISPE-HPLC-UV method was developed for the determination of SMO and SDZ in egg and milk samples. Acrylamide-functionalized silica nanoparticles (50 mg), SMO (50 mg), AM (40 mg), EGDMA (3 mL) and AIBN (30 mg) were dissolved in acetonitrile (50 mL) and the mixture was sonicated for 10 min and left at room temperature for 3 h. Polymerization was carried out under nitrogen stream and continuous stirring at 60 °C for 24 h and the mixture was left to age at 85 °C for 3 h. The developed MIP nanoparticles were washed with acetonitrile and the template was removed by Soxhlet extraction with methanol-acetic acid (9:1, *v*/*v*). The MIP nanoparticles were washed methanol and dried under vacuum at 60 °C. SMO-MIP nanoparticles (100 mg) were added to pretreated spiked milk extract (50 mL) and the mixture was incubated for 45 min at room temperature and centrifuged at 3000 rpm for 10 min. The supernatant was discarded and the nanoparticles were collected. The analytes were eluted with methanol-acetic acid (9:1, *v*/*v*), the elute was evaporated under nitrogen stream and the dry residue was reconstituted with methanol (1 mL). In comparison with the respective NIP, MIP adsorption capacity for SMO was higher, with Q_max_ values of 5634 and 20,210 and μg/g, while binding specificity study between SMO, SDZ, SMZ, sulfadimethoxine, sulfamerazine and sulfameter showed MIP selectivity over SMO and the imprinting factor was 21.52 for SMO. The SiO_2_-SMO-MIP nanoparticles were compared with SMO-MIPs prepared by bulk polymerization in terms of SMO binding capacity and demonstrated three times higher capacity. Furthermore, SMO adsorption equilibrium was achieved in 45 min with the SiO_2_-SMO-MIP nanoparticles and 10 h with the SMO-MIPs, proving that the developed MIP nanoparticles had higher number of imprinted sites and improved accessibility by the analytes [85].

A MSPE-HPLC-UV method was developed for the determination of SMO in milk and honey samples. SMO (150 mg) and MAA (225 μL) were dissolved in acetonitrile (30 mL) and the mixture was stored for 12 h in a refrigerator. A second mixture was prepared by dissolving vinyl-modified magnetic carbon nanotubes (CNTs) (100 mg) in acetonitrile (30 mL), EGDMA (1 mL) and AIBN (40 mg) were added, and the mixture was sonicated 30 min and degassed under nitrogen stream for 10 min. The two mixtures were combined and stirred and left to pre-polymerize at 50 °C for 6 h, polymerize at 60 °C for 24 h and left to age at 75 °C for 6 h under nitrogen stream and continuous stirring. The template was removed by incubating the developed MIPs in methanol-acetic acid (9:1, *v*/*v*) and the MIP particles were washed with methanol and dried under vacuum. Magnetic CNTs-SMO-MIPs (100 mg) were added into pretreated spiked milk extract (20 mL) and the mixture was kept for 1 h. The MMIPs were collected by means of a magnet and the supernatant was discarded. The analytes were eluted with methanol-acetic acid (9:1, *v*/*v*), the eluate was reduced and the residue was reconstituted with acetonitrile (1 mL). In comparison with the respective NIP, MIP adsorption capacity for SMO was higher, with Q_max_ values of 864.9 μg/g, while binding specificity study between SMO, SMZ, sulfadimethoxine, sulfamerazine and sulfameter showed MIP selectivity over SMO and the imprinting factors were 10 for SMO, 1.7 for SMZ, 1.9 for sulfadimethoxine, 2.3 for sulfamerazine and 1.5 for sulfameter [86].

A PMME-HPLC-PAD method was developed for the determination of SMO. SMO (0.05 mmol) dissolved in acetonitrile (400 μL) was mixed with AM (0.1 mmol) and 4-VP (0.1 mmol) and mixture was sonicated for 4 h. EGDMA (1 mmol) and AIBN (9.7 mg) were added and was degassed under nitrogen stream for 10 min. The mixture (50 μL) was transferred and sealed inside a micropipette tip and polymerization was carried out at 60 °C for 24 h. 

The template was removed by incubation in methanol. The developed MIP monolith containing micropipette tip was connected to a syringe that was employed for the sample loading, washing and elution steps. The SMO-MIP monolith was preconditioned with acetonitrile (2 mL) and water (1 mL), loaded with pretreated milk extract (5 mL) and washed with water (0.5 mL). The analytes were eluted with acetonitrile (0.1 mL) and the eluate was injected directly for analysis. The developed magnetic SMO-MIPs could be reused for five extraction cycles [87].

A SBSE-HPLC-UV method was developed for the determination of trimethoprim, SMZ, sulfamerazine and sulfameter in urine, plasma and milk samples. Trimethoprim (290.4 g) and MAA (0.34 mL) were dissolved in acetonitrile (20 mL) and the mixture was stored for 12 h at room temperature. EGDMA (2.26 mL) and AIBN (90 mg) were added and the mixture was degassed under sonication for 5 min. A silylated glass capillary was introduced into the mixture (1.5 mL) inside a test tube and polymerization was carried out at 60 °C for 2 h. The procedure was repeated for seven times until a coating of 21.5 μm thickness was acquired. A magnetic core was sealed inside the glass capillary which was then coated with a 2-cm thick coating. The template was removed by incubating the developed MIP coated stir bars in methanol-acetic acid (9:1, *v*/*v*). A trimethoprim-MIP coated stir bar was introduced in pretreated spiked milk extract (10 mL) and extraction was carried out under stirring at 500 rpm for 45 min. The stir bar was collected and analytes were eluted with acetonitrile-phosphoric acid (20:80, *v*/*v*) and sonication for 2 min. The extraction capacity of the MIP-coating was found 1.7 times higher than the NIP-coating [88]. All the reported applications are summarized in Table 5.

## 6. Conclusions

This review has successfully reported novel applications of MIPs as sorbent materials for the extraction and chromatographic determination of various antibiotic categories in milk samples. Overall, MIP-based techniques offer improved sample clean-up efficiency and increased selectivity over the target analytes. The reported methods achieved lower limits of detection and quantification (LOD and LOQ) or decision limits and detection capabilities (CC_α_ and CC_β_) and equally good extraction recoveries compared with methods that employ sample preparation techniques with conventional sorbent materials (Table 6). Furthermore, in many cases MIPs could be reused for more than one extraction cycle in contrast with conventional materials that have only one use. However, MIPs are mainly applied in deproteinized and defatted milk extracts and not directly in milk samples, for the extraction and determination of structurally related analytes with the template molecule used for their synthesis. Additionally, template removal requires increased organic solvent amount and is a rather time-consuming procedure. There is no deny of the usefulness of the MIPs in sample preparation. MIPs future perspectives include improved MIP synthesis and efficient template removal, MIPs with multi-class and multi-analyte extraction capability, as well as direct sample application.

## Figures and Tables

**Table 1:** MIPs applications for the determination of amphenicols in milk samples.

Analyte	Matrix	MIP Composite	Polymerization Technique	Template-Monomer-Cross Linker	Extraction-Analysis	LOD, LOQ–CC_α_, CC_β_	Recoveries (%)	Ref.
CAP	milk	TAP-MIPs	precipitation polymerization	TAP-MAA-EGDMA	MISPE-LC-MS/MS	LOD (μg/kg): 0.02 LOQ (μg/kg): 0.08	96.04–108.68	[48]
CAP	milk and shrimps	CAP-MIP microspheres	suspension polymerization	CAP-DEAEM-EGDMA	MISPE-HPLC-UV	-	90.2–99.9	[49]
CAP	milk	CAP-MIPs	sol-gel synthesis	CAP-TEPS and 3-APTES (functional precursors)-TMOS	MISPE-LC-MS	LOD (μg/kg): 0.1 LOQ (μg/kg): 0.3	85–106	[50]
CAP	milk and honey	CAP-chitosan-MIPs	-	CAP-AM-EGDMA	MISPE-HPLC-DAD	-	94.96–95.20	[51]
FFC	milk	FFC-MIPs	emulsion polymerization	FFC-AM-EGDMA	MISPE-LC-MS/MS	LOD (μg/L): 4.1 LOQ (μg/L): 13.9	88.7–93.8	[52]
CAP, TAP, FFC	baby formulas	CAP-MIPs	precipitation polymerization	CAP-MAA-DVB	MISPE-HPLC-MS/MS	CC_α_ (μg/L): 0.06–10.5 CC_β_ (μg/L): 0.1–18	91–110	[53]
TAP	milk and honey	TAP-MIP monoliths	-	TAP-4-VP-EGDMA	PMME-HPLC-DAD	LOD (μg/L): 3	92.9–97.5	[54]
TAP	milk and honey	CAP-MIP monoliths	-	CAP-4-VP-EGDMA	PMME-HPLC-DAD	LOD (μg/kg): 5 LOQ (μg/kg): 17	93.5–96.8	[55]
CAP	raw milk, skimmed milk and milk powder	MIP4SPE-CAP cartridges	-	-	MISPE-LC-ESI-MS/MS	CC_α_ (μg/kg): 0.06 CC_β_ (μg/kg): 0.10	50–87	[56]
CAP	honey, urine, milk (raw and semi-skimmed) and plasma	SupelMIP-CAP SPE cartridges	-	-	MISPE-HPLC-UV	CC_α_ (μg/kg): 0.02 CC_β_ (μg/kg): 0.03	67–82.3	[57]
CAP	urine, feed water, cow milk and honey	SupelMIP-CAP SPE cartridges	-	-	MISPE-GC-NCI-MS	CC_α_ (μg/L): 0.03 CC_β_ (μg/L): 0.05	-	[58]

**Table 2:** MIPs applications for the determination of penicillins in milk samples.

Analyte	Matrix	MIP Composite	Polymerization Technique	Template-Monomer-Cross Linker	Extraction-Analysis	LOD, LOQ–CC_α_, CC_β_	Recoveries (%)	Ref.
AMP	cow milk	AMP-MIPs	bulk polymerization	AMP-MAA-EGDMA	MISPE-HPLC-UV	LOD (μg/L): 10.7 LOQ (μg/L): 35.8	>95	[59]
benzylpenicillin	raw milk	benzylpenicillin-MIPs	suspension polymerization	benzylpenicillin-MAA-TRIM	MISPE-LC-MS/MS	LOD (μg/kg): 0.51 LOQ (μg/kg): 1.02	70–110	[60]
OXA, CLOX, DICLOX	milk	2-biphenylylpenicillin-MIPs	-	2-biphenylylpenicillin-EAMA-TRIM	MISPE-HPLC-DAD	LOD (μg/kg): 1.6–1.9 LOQ (μg/kg): 5.3–6.3	94–101	[61]
AMP, AMX, OXA, PEN G, PEN V, CLOX, DICLOX, NAFC	baby formulas	NAFC-MIPs	precipitation polymerization	NAFC-MAA-EGDMA	MISPE-HPLC-MS/MS	LOD (μg/kg): 0.9–23.6 LOQ (μg/kg): 3.1–78.4	60–91	[62]
PEN V, AMX, OXA	milk	PEN V-MMIPs	-	PENV-MAA-EGDMA	MSPE-LC-MS/MS	-	70.3–78.6	[63]
penicilloic acid, penilloic acid	milk	penicilloic acid-SMIPs	-	penicilloic acid-MAA-EGDMA	MSPD-HPLC-UV	LOD (μg/g): 0.04–0.05 LOQ (μg/g): 0.13–0.17	77.4–90.3	[64]

**Table 3:** MIPs applications for the determination of quinolones in milk samples.

Analyte	Matrix	MIP Composite	Polymerization Technique	Template-Monomer-Cross Linker	Extraction-Analysis	LOD, LOQ–CC_α_, CC_β_	Recoveries (%)	Ref.
FLX, ENO, PEF, NOR, CIP, LVFX, LOM, ENR, GAT, SPA	milk	MTMIPs	bulk polymerization	LVFX and CIP-MAA-TRIM	MISPE-HPLC-DAD	LOD (μg/kg): 1.95–7.35 LOQ (μg/kg): 4.45–24.49	84.1–104.7	[65]
estriol, estrone, 17β-estradiol, OFL, NOR, CIP	milk	MTMIPs	-	OFL and 17β-estradiol-MAA-EGDMA	MISPE-HPLC-UV	-	86.7–94.8	[66]
OFL, LOM, CIP, ENR	milk	ENR-MIP-HCM column	sol-gel synthesis	ENR-MAA and HEMA-EGDMA	on-line MISPE-HPLC-UV	LOD (μg/kg): 1.37–3.74 LOQ (μg/kg): 4.56–12.5	89.1–99.2	[67]
CIP, DIF, DAN, ENR	milk	PEF-MIP monoliths	in situ polymerization	PEF-MAA-DEGDMA	PMME-HPLC-FLD	LOD (μg/L): 0.4–1.6 LOQ (μg/L): 2.7–4.7	92.4–96.5	[68]
OFL, NOR, ENR	milk	hollow OFL-MIPs	-	OFL-MAA-EGDMA	DSPE/HPLC–UV	LOQ (μg/L): 20–30	90.9–102.6	[69]
OFL, CIP, LOM	milk	MMIPs	-	-	MSPE-HPLC-UV	LOD (μg/kg): 1.8–3.2 LOQ (μg/kg): 6.1–10.8	101.6–124.4	[70]

**Table 4:** MIPs applications for the determination of tetracyclines in milk samples.

Analyte	Matrix	MIP Composite	Polymerization Technique	Template-Monomer-Cross Linker	Extraction-Analysis	LOD, LOQ–CC_α_, CC_β_	Recoveries (%)	Ref.
CTC, TC, OTC, DC	milk, egg and pork	CTC-MIPs	precipitation polymerization	CTC-MAA-EGDMA	MISPE-HPLC-DAD	LOD (μg/L): 20–40 LOQ (μg/L): 50–80	77.5–93	[71]
TC, CTC, DC	milk	DC-MIP-HCMs	precipitation polymerization	DC-MAA-DVB	MISPE-HPLC-UV	LOD (μg/kg): 7500–13,800 LOQ (μg/kg): 24,700–46,000	85–106	[72]
DC, TC, CTC	milk and honey	DC-MIP-HCMs	sol-gel synthesis	DC-MAA- TEOS (inorganic precursor) and KH570 (coupling agent)	MISPE-HPLC-UV	LOD (μg/kg): 4.9–15.3 LOQ (μg/kg): 16.6–51	74.7–115.5	[73]
OTC, MTC, DC	milk	OTC-MIP-HCMs	sol-gel synthesis	OTC-MAA-TEOS (inorganic precursor) and KH570 (coupling agent)	MISPE-HPLC-UV	LOD (μg/kg): 4.8–12.7 LOD (μg/kg): 16–42.3	80.9–104.3	[74]
TC, OTC, DMC	egg, milk, and milk powder	TC-MIP-HCMs	sol-gel synthesis	TC-MAA-TEOS (inorganic precursor) and KH570 (coupling agent)	on-line MISPE-HPLC-UV	LOD (μg/kg): 0.76–1.13 LOQ (μg/kg): 2.53–3.77	85.9–98.3	[75]
TC, OTC, CTC, MC	milk	MC-imprinted poly(MAA-g- MAPS-GO-MBA) monolith	-	MC-MAA-MBA	PMME-HPLC-UV	LOD (μg/L): 30–53 LOQ (μg/L): 100–176	83.7–109.3	[76]
OTC, TC, DC, CTC	chicken feed, chicken muscle and milk	TC-MIPs coating	-	TC-AM-TRIM	SPME-HPLC-FLD	LOD (μg/L): 1.02–2.31	75.7–93.7	[77]
OTC, TC, CTC, DC	milk	DC-MIMMs	suspension polymerization	DC-MAA and AM-TRIM	MDE-HPLC-UV	LOD (μg/kg): 7.4–19.4 LOQ (μg/kg): 24.7–64.7	74.5–93.8	[78]
OTC, TC, CTC, DC	milk	CTC-MIMMs	suspension polymerization	CTC-MAA and AM-TRIM	MDE-HPLC-UV	LOD (μg/kg): 5.71–11.18 LOQ (μg/kg): 19.02–37.28	76.4–95.84	[79]

**Table 5:** MIPs applications for the determination of cephalosporins, macrolides and sulfonamides in milk samples.

Analyte	Matrix	MIP Composite	Polymerization Technique	Template-Monomer-Cross Linker	Extraction-Analysis	LOD, LOQ–CC_α_, CC_β_	Recoveries (%)	Ref.
CFL	milk	CFL-MIPs	bulk polymerization	CFX-TFMAA-EGDMA	MISPE-HPLC-UV	-	91.78–93.25	[80]
CFL, CFP	milk	tributylammonium CFD-MIPs	-	tributylammonium CFD-MAA-EGDMA	MISPE-HPLC-UV	-	>60	[81]
THIO, CFZ, ALO, CFP, QUI, CFL	milk	sodium 7-(2-biphenylylcarboxamido)-3-methyl-3-cepheme-4-carboxylate-MIPs	-	sodium 7-(2-biphenylylcarboxamido)-3-methyl-3-cepheme-4-carboxylate-VPU-DVB	MISPE-UHPLC-MS/MS	LOD (μg/kg): 0.1–3.8 LOQ (μg/kg): 0.4–12.5	15–100	[82]
SPI	sheep milk	SPI-MIPs	bulk polymerization	SPI-MAA-EGDMA	MISPE-HPLC-DAD	LOQ (μg/kg): 24.1	>90	[83]
ERY	sheep milk	ERY-MIPs	bulk polymerization	ERY-MAA-EGDMA	MISPE-HPLC-DAD	LOQ (μg/kg): 24.1	>98	[84]
SMO, SDZ	eggs and milk	SMO-MIPs	-	SMO-AM-EGDMA	MISPE-HPLC-UV	LOD (μg/L): 2.81–8.21	69.8–87.4	[85]
SMO	milk and honey	Magnetic CNTs-SMO-MIPs	-	SMO-MAA-EGDMA	MSPE-HPLC-UV	LOD (μg/L): 6.04	68.3–78.2	[86]
SMO	milk	SMO-MIP monoliths	-	SMO-AM and 4-VP-EGDMA	PMME-HPLC-PAD	LOD (μg/L): 1	93.6–101.7	[87]
trimethoprim, SMZ, sulfamerazine, sulfamether	urine, plasma and milk	trimethoprim-MIPs coating	-	trimethoprim-MAA-EGDMA	SBSE-HPLC-UV	LOD (μg/L): 3.2–4.8	83.2–110.2	[88]

**Table 6:** MIPs applications and conventional extraction techniques.

Analyte	Matrix	Extraction-Analysis	LOD, LOQ–CC_α_, CC_β_	Recoveries (%)	Ref.
TAP	milk and honey	PMME-HPLC-DAD	LOD (μg/kg): 5 LOQ (μg/kg): 17	93.5–96.8	[55]
CAP	honey, urine, milk (raw and semi-skimmed) and plasma	MISPE-HPLC-UV	CC_α_ (μg/kg): 0.02 CC_β_ (μg/kg): 0.03	67–82.3	[57]
amphenicols: CAP, TAP, FFC penicillins: AMP, AMX, OXA, CLOX, DICLOX	milk	MSPD-HPLC-DAD	LOD (μg/kg): 11–15 (amphenicols), 6–12 (penicillins) LOQ (μg/kg): 35–45 (amphenicols), 20–46 (penicillins) CC_α_ (μg/kg): 48.4–56.3 (amphenicols), 35.2–57.2 (penicillins) CC_β_ (μg/kg): 52.7–61.4 (amphenicols), 39.9–61.9 (penicillins)	85–94 (amphenicols) 84–85 (penicillins)	[89]
THIO, CFZ, ALO, CFP, QUI, CFL	milk	MISPE-UHPLC-MS/MS	LOD (μg/kg): 0.1–3.8 LOQ (μg/kg): 0.4–12.5	15–100	[82]
AMP	cow milk	MISPE-HPLC-UV	LOD (μg/L): 10.7 LOQ (μg/L): 35.8	>95	[59]
AMP, AMX, OXA, PEN G, PEN V, CLOX, DICLOX, NAFC	baby formulas	MISPE-HPLC-MS/MS	LOD (μg/kg): 0.9–23.6 LOQ (μg/kg): 3.1–78.4	60–91	[62]
cephalosporins: CFL, THIO, cefaclor, cefadroxil, cefuroxime, cefoperazone, cefotaxime, cefazolin	milk	MSPD-HPLC-DAD	CC_α_ (μg/kg): 53.94–54.35, 105.25–113.31 CC_β_ (μg/kg): 54.4–56.3, 103.5–112.3	93.4–108.6	[90]
cephalosporins: CFL, THIO, cefaclor, cefadroxil, cefuroxime, cefoperazone, cefotaxime, cefazolin penicillins: AMX, OXA, CLOX, DICLOX	milk	MSPD-HPLC-DAD	LOD (μg/kg): 6.3–15.1 (cephalosporins), 6.7–15.3 (penicillins) LOQ (μg/kg): 19.2–45.7 (cephalosporins), 20.3–46.5 (penicillins) CC_α_ (μg/kg): 54–111 (cephalosporins), 35–38 (penicillins) CC_β_ (μg/kg): 61–122 (cephalosporins), 38–42 (penicillins)	85–92 (cephalosporins) 82–90 (penicillins)	[91]
FLX, ENO, PEF, NOR, CIP, LVFX, LOM, ENR, GAT, SPA	milk	MISPE-HPLC-DAD	LOD (μg/kg): 1.95–7.35 LOQ (μg/kg): 4.45–24.49	84.1–104.7	[65]
CIP, DIF, DAN, ENR	milk	PMME-HPLC-FLD	LOD (μg/L): 0.4–1.6 LOQ (μg/L): 2.7–4.7	92.4–96.5	[68]
cephalosporins: CFL, cefaclor, cefadroxil, cefuroxime, cefoperazone, cefotaxime, ceftiofur, cefazolin quinolones: CIP, DAN, ENO, ENR, NOR, OFL, OXO, sarafloxacin, flumequine, nalidixic acid	milk	MSPD-LC-MS/MS	LOQ (μg/kg): 2.4–15	81.7–114.9 (quinolones) 81.4–117 (cephalosporins)	[92]
quinolones: CIP, DAN, ENO, ENR, NOR, OFL, OXO, sarafloxacin, flumequine, nalidixic acid	milk	SPE-HPLC-DAD	LOD (μg/L): 1.5–6.8	75–92	[93]
SMO, SDZ	eggs and milk	MISPE-HPLC-UV	LOD (μg/L): 2.81–8.21	69.8–87.4	[85]
sulfonamides: SMO, SDZ, sulfathiazine, sulfamethoxine, sulfamethizole, sulfamethoxypyridazine, sulfamonomethoxine, sulfisoxazole, sulfadimethoxine, sulfaquinoxaline	milk	LLE-HPLC-DAD	LOD (μg/kg): 2.3–9.7 LOQ (μg/kg): 7–29.2 CC_α_ (μg/kg): 101.61–108.11 CC_β_ (μg/kg): 105.64–119.01	93.9–115.9	[94]
OTC, MTC, DC	milk	MISPE- HPLC-UV	LOD (μg/kg): 4.8–12.7 LOD (μg/kg): 16–42.3	80.9–104.3	[74]
OTC, TC, CTC, DC	milk	MDE-HPLC-UV	LOD (μg/kg): 5.71–11.18 LOQ (μg/kg): 19.02–37.28	76.4–95.84	[79]
tetracyclines: MC, TC, OTC, MTC, DMC, CTC, DC	milk	SPE-HPLC-DAD	CC_α_ (μg/kg): 101.25–105.84 CC_β_ (μg/kg): 103.94–108.88	93.8–107.2	[95]
tetracyclines: OTC, TC, CTC, DC, epi-chlorotetracycline	milk	MSPD-HPLC-DAD	LOD (μg/kg): 4.8–18.7 LOQ (μg/kg): 14.5–56.6 CC_α_ (μg/kg): 62–113 CC_β_ (μg/kg): 70.8–115	82–108	[96]
